# Excess filaggrin in keratinocytes is removed by extracellular vesicles to prevent premature death and this mechanism can be hijacked by *Staphylococcus aureus* in a TLR2‐dependent fashion

**DOI:** 10.1002/jev2.12335

**Published:** 2023-06-20

**Authors:** Adrian Kobiela, Lilit Hovhannisyan, Paulina Jurkowska, Jorge Bernardino de la Serna, Aleksandra Bogucka, Milena Deptuła, Argho Aninda Paul, Kinga Panek, Ewa Czechowska, Michał Rychłowski, Aleksandra Królicka, Jacek Zieliński, Susanne Gabrielsson, Michał Pikuła, Magdalena Trzeciak, Graham S Ogg, Danuta Gutowska‐Owsiak

**Affiliations:** ^1^ Experimental and Translational Immunology Group Intercollegiate Faculty of Biotechnology of University of Gdansk and Medical University of Gdansk, University of Gdansk Gdansk Poland; ^2^ National Heart and Lung Institute Imperial College London London UK; ^3^ Central Laser Facility, Rutherford Appleton Laboratory, Medical Research Council‐Research Complex at Harwell Science and Technology Facilities Council UK; ^4^ The Mass Spectrometry Laboratory Intercollegiate Faculty of Biotechnology of University of Gdansk and Medical University of Gdansk Gdansk Poland; ^5^ Laboratory of Tissue Engineering and Regenerative Medicine, Department of Embryology Medical University of Gdansk Gdansk Poland; ^6^ Laboratory of Virus Molecular Biology Intercollegiate Faculty of Biotechnology of University of Gdansk and Medical University of Gdansk, University of Gdansk Gdansk Poland; ^7^ Laboratory of Biologically Active Compounds Intercollegiate Faculty of Biotechnology of University of Gdansk and Medical University of Gdansk, University of Gdansk Gdansk Poland; ^8^ Department of Surgical Oncology Medical University of Gdansk Gdansk Poland; ^9^ Division of Immunology and Allergy Department of Medicine Solna, Karolinska Institutet Stockholm Sweden; ^10^ Department of Clinical Immunology and Transfusion Medicine Karolinska University Hospital Stockholm Sweden; ^11^ Department of Dermatology Venereology and Allergology, Medical University of Gdansk Gdansk Poland; ^12^ Medical Research Council (MRC) Human Immunology Unit, Medical Research Council (MRC) Weatherall Institute of Molecular Medicine National Institute for Health Research (NIHR) Oxford Biomedical Research Centre, Radcliffe Department of Medicine, University of Oxford Oxford UK

**Keywords:** atopic dermatitis, bacteria, exosomes, extracellular vesicles, filaggrin, keratinocyte, *Staphylococcus aureus*

## Abstract

Filaggrin (FLG) protein is indispensable for multiple aspects of the epidermal barrier function but its accumulation in a monomeric filaggrin form may initiate premature keratinocytes death; it is unclear how filaggrin levels are controlled before the formation of storing keratohyalin granules. Here we show that keratinocyte‐secreted small extracellular vesicles (sEVs) may contain filaggrin‐related cargo providing a route of eliminating excess filaggrin from keratinocytes; blocking of sEV release has cytotoxic effects on those cells. Filaggrin‐containing sEVs are found in plasma in both healthy individuals and atopic dermatitis patients. *Staphylococcus aureus (S. aureus)* enhances packaging and secretion of filaggrin‐relevant products within the sEVs for enhanced export via a TLR2‐mediated mechanism which is also linked to the ubiquitination process. This filaggrin removal system, preventing premature keratinocyte death and epidermal barrier dysfunction, is exploited by *S. aureus* which promotes filaggrin elimination from the skin that could help safeguard bacterial growth.

## Graphical abstract

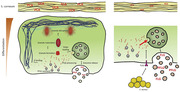

## 1 INTRODUCTION

A functional epidermal barrier is critical for survival as it protects an organism from the environmental impact. The process of its formation is complex and depends on complete keratinocyte differentiation promoting acquisition of unique cellular functions by these cells and eventual cell death by cornification, leading to *stratum corneum* formation (Eckhart et al., 2013). While multiple mechanisms are involved (Eyerich et al., 2018), one remarkable protein, filaggrin (FLG) (Brown & Irwin Mclean, 2012) proved paramount; *FLG* mutation(s) (Palmer et al., 2006) confer the highest inherited risk for atopic dermatitis (AD). Several barrier‐supporting functions of the protein have been documented, from the mechanical strengthening of the corneocytes through to effect on immunity (Lee et al., 2010; Leitch et al., 2016; Marwah et al., 2014) and indirect antimicrobial activity (Mcaleer & Irvine, 2013). Filaggrin knockdown models show deep abnormality in keratinocyte differentiation process, including the effect on metabolic pathways (Elias et al., 2019; Wang et al., 2017) and enzymatic activity (Wang et al., 2017) corresponding to the findings from AD skin. Filaggrin's role is especially apparent upon disintegration of profilaggrin (PFLG)‐storing keratohyalin granules (KHGs); subsequent accumulation of filaggrin monomers in the cytosol leads to collapse of the keratin‐based intermediate filaments (IFs), disruption of cellular junctions and initiation of programmed cell death (Presland et al., 2001). While those effects are in line with the physiological filaggrin function at the verge of keratinocyte's life, given the irreversibility of this process, filaggrin containment and release must be tightly controlled to avoid premature cell death and failure of stratification (Presland et al., 2001). We have previously described a mechanism involving KHG‐associated actin scaffolds controlling filaggrin sequestration and its coordinated release in terminally differentiated keratinocytes (Gutowska‐Owsiak et al., 2018); the process requires prior activation of an independent differentiation‐linked AKT1‐HspB1 pathway (O'haughnessy et al., 2007). Recently, a complementary mechanism, describing KHGs as biomolecular condensates formed by liquid phase separation has been also proposed (Quiroz et al., 2020). Here we show evidence of low dispersed cytoplasmic level of profilaggrin/filaggrin in keratinocytes before the appearance of KHGs, and demonstrate that filaggrin‐related products are loaded as cargo into exosome‐enriched keratinocyte‐derived small extracellular vesicles (sEVs). We further show that *Staphylococcus aureus* (*S. aureus*), frequently colonising the atopic skin (Nakatsuji et al., 2016) exploits this mechanism which leads to dysregulation of sEV production in keratinocytes and enhanced removal of filaggrin from the cell.

## 2 RESULTS

### 2.1 Keratinocytes express low levels of cytoplasmic filaggrin early during differentiation

**FIGURE 1:**
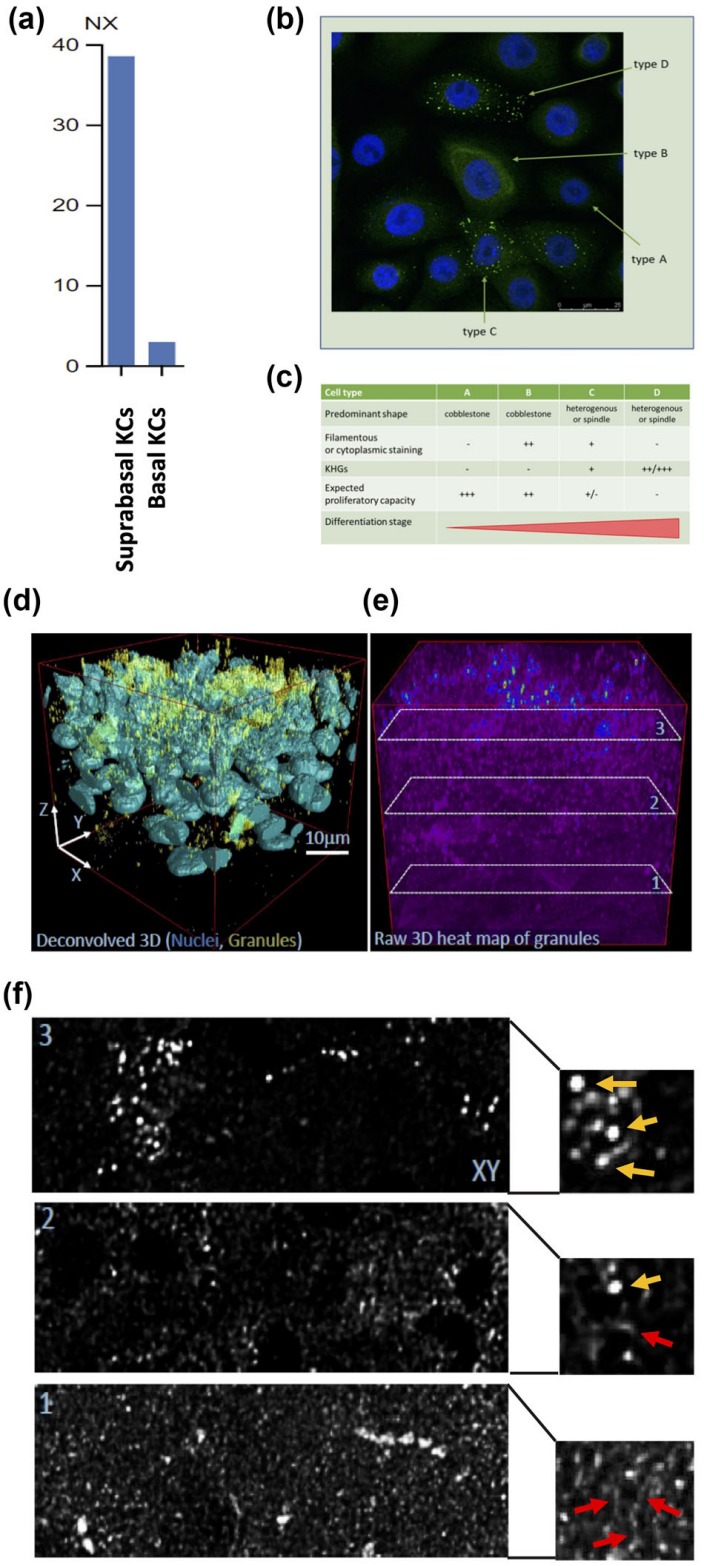
Low levels of cytoplasmic filaggrin are expressed in keratinocytes during early differentiation, before appearance of keratohyalin granules. (a) Filaggrin mRNA expression in primary keratinocytes (KCs), data extracted from ProteinAtlas (Karlsson et al., 2021; Uhlén et al., 2015; Uhlén et al., 2019); (b) example immunofluorescence image of filaggrin protein expression patterns in primary keratinocytes (*n* = 3); nucleus: blue; filaggrin: green (c) comparison of primary keratinocyte features and filaggrin expression by cells at various differentiation stages; (d)–(e) representative image of whole epidermal sheets (*n* = 5); (d) deconvolved image, indicating KHG accumulation; (e)–(f) representative cross‐section of epidermal sheet (*n* = 6) shown in (d); (f) close‐up panels indicating filamentous staining suggestive of IFs (red arrows) predominantly in the lower layers and granular pattern of staining indicating KHGs (yellow arrows), enriched in the upper layers.

Filaggrin expression in keratinocytes increases significantly during differentiation as the result of the positive feedback loop initiated by nuclear signalling of the profilaggrin N‐terminal domain (Ishida‐Yamamoto et al., 1998; Pearton et al., 2002). As such, the expression pattern is expected to follow the model of expression dynamics for proteins undergoing positive autoregulation (Mitrophanov & Groisman, 2008), with synthesis gradually increasing. There is evidence that a substantial local filaggrin concentration threshold must be reached (Quiroz et al., 2020) before KHGs may form; before this happens, however, transcriptional bursting (Wotherspoon et al., 2020) would give rise to low levels of RNA expression. Accordingly, *FLG* mRNA transcript can be detected already in basal epidermal keratinocytes (Protein Atlas Karlsson et al., 2021; Uhlén et al., 2015, 2019; Figures 1a and S1) (Lee et al., 2020). The stochastic nature of transcriptional bursting suggests that this may be possible also in cells other than keratinocytes, for example, fibroblasts, immune cells and cell lines of a diverse origin; filaggrin transcript can be detected in those too, albeit at low levels (Figure S1). Local protein levels can be much higher in keratinocytes, however, and reach the threshold for KHG formation; we hypothesise that initially, before the threshold is met, unsequestered profilaggrin molecules escape into the cytosol, where they may undergo processing and IF binding.

To investigate this, we assessed profilaggrin/filaggrin expression in normal human epidermal keratinocytes (NHEKs); depending on the differentiation advancement these cells showed a continuum between cytoplasmic/filamentous and granular filaggrin staining distribution (Figure 1b,c). Specifically, we found proliferatory NHEKs of a cobblestone morphology either negative or showing low/medium intensity of cytoplasmic or filamentous staining and no KHG expression. In contrast, more differentiated keratinocytes showed a degree of ‘dot positivity’ indicative of KHG formation but had either very little or no cytoplasmic/filamentous staining. Finally, we also inspected stratified epidermis and observed low level of cytoplasmic/filamentous staining in the lower cell layers and complete disappearance of such signal upon the appearance of KHGs (Figures 1d–f, S2).

### 2.2 Profilaggrin/filaggrin segregate into small EVs secreted by keratinocytes

Given that keratinocytes that are of low differentiation status already express potentially cytotoxic filaggrin monomers, we considered presence of an active mechanism allowing keratinocytes to efficiently remove excess unsequestered profilaggrin/filaggrin molecules. Here, we hypothesised that the cells may employ small extracellular vesicles (sEVs) such as exosomes, for this purpose. Hence, to get some initial indication if profilaggrin/filaggrin segregate into EVs we first carried out a search in the manually curated EV database (Vesiclepedia; available at http://microvesicles.org/) (Kalra et al., 2012) under terms ‘filaggrin’ and ‘FLG’, complemented with the manual PubMed search (the phrase ‘filaggrin’ or ‘profilaggrin’ and ‘exosomes’ or ‘microvesicles’ or ‘apoptotic bodies’ or ‘extracellular vesicles’). The results identified several datasets reporting mass spec signal indicative of the presence of profilaggrin/filaggrin‐derived peptides in EVs from different sample sources, mostly within the sEV/exosomal fractions. As expected, the protein was mainly reported in samples for which epithelial cells were the most probable source of EVs, for example, urine, breast milk, nasal lavage, thymic epithelial cell and epithelial cancer cultures (Table 1); however, interestingly signal was also reported in T lymphoblast‐derived exosomes, which would be in line with Protein Atlas (Uhlén et al., 2015) RNA expression data (Figure S1).

**TABLE 1:** Studies reporting the presence of profilaggrin/filaggrin‐related peptides in extracellular vesicle fractions, as reported in Vesiclepedia, complemented with manual search in Pubmed. MV, microvesicles; Exo, exosomes; MP, microparticles.

Source	EV	Isolation methods	Markers	PubMed	FBS info
**Human colorectal cancer ascites**	MV	Serial centrifugations Sucrose cushion UC (100,000 x *g*) OptiPrep density gradient UC (200,000 x *g*)	Alix, CD81, Tsg101, β‐actin, ICAM‐1, Ezrin, Catenin β−1	21630462	n/a
**Breast milk**	EV	Sucrose gradient UC (192,000 x *g*)	CD9, Annexin A5, Flotillin‐1, OLAH and PTHLH—specific to EV derived from milk	27601599	n/a
**Thymic epithelial culture**	Exo	UC (100,000 x *g*)	TSG101, HLA‐DR, CD9 and CD81, negative for CD45 and close to negative for CD63	25776846	n/a
**Human bone marrow mesenchymal stem cells**	MV	UC Ultrafiltration Sucrose cushion UC (100,000 x *g*) OptiPrep density gradient UC (200,000 x *g*) UC wash (100,000 x *g*)	CD63, β‐actin, HSP90, galectin‐1	22148876	10% FBS (no information regarding depletion)
**Platelet‐poor plasma**	MP	Centrifugation (18,890 x *g*) Centrifugation washes 2, 4, 6, 8, and 10 times	ITGA2B, transthyretin, actin, HSA	22329422	n/a
**Plasma of coronary artery disease patients**	MP	Centrifugation (19,000 x *g*) Centrifugation wash	CD31, CD41, CD62E, CD146, CD14	23056467	n/a
**Plasma**	Exo	Exo‐SpinTM Blood kit Size exclusion chromatography	CD9, CD81, CD5L, LGALS3BP	26154623	n/a
**Cultured human renal proximal tubule cells**	Exo	UC (200,000 x *g*) Immunomagnetic isolation with anti‐CD63 coupled to magnetic MPs	TSG101	24976626	10% FBS (no information regarding depletion)
**Seminal plasma**	Prostasomes	UC (100,000 x *g*) xK16/70 Superdex 200 gel column Sucrose gradient UC (85,000 x *g*) UC wash (100,000 x *g*)	Flotillin‐1, Flotillin‐2, Clathrin, Caveolin‐1, Caveolin‐2	26272980	n/a
**Donor primary lymphoblast** **cells**	EV	UC (100,000 x *g*)	ERM, filamin, α‐actinin, nucleolin, β‐actin, Rac1/2, EF‐1α	23463506	10% FBS (depleted by overnight centrifugation at 100,000 x *g*)
**Urine**	Exo	UC (200,000 x *g*) Sucrose (17,000 x *g*) UC (200,000 x *g*)	–	22106071	n/a
**Urine**	Exo	Differential centrifugation (200,000 x *g*)	–	22641613	n/a
**Human amnion epithelial cells**	Exo	Differential UC (100,000 x *g*)	CD9, CD63, CD81, HSC70	27333275	10% Exo‐free FBS (System Biosciences, Mountain View, CA)
**Nasal lavage fluid**	Exo	Differential centrifugation (120,000 x *g*)	CD63, CD9 and TSG101	27320496	n/a
**Human Amnion Epithelial Cells**	EV	ExosomeSerial centrifugation UC (100,000 x *g*)	CD9, CD63, CD81 and HSC70	27333275	n/a
**Human adipose‐derived mesenchymal stem cells**	Exo	Differential centrifugation UC 100,000 x *g*	CD9 and CD63	33933157	n/a
**Human sweat**	Exo	Differential centrifugation UC 100,000 x *g*	Alic, CD63, Hsp70	28899687	n/a

**FIGURE 2:**
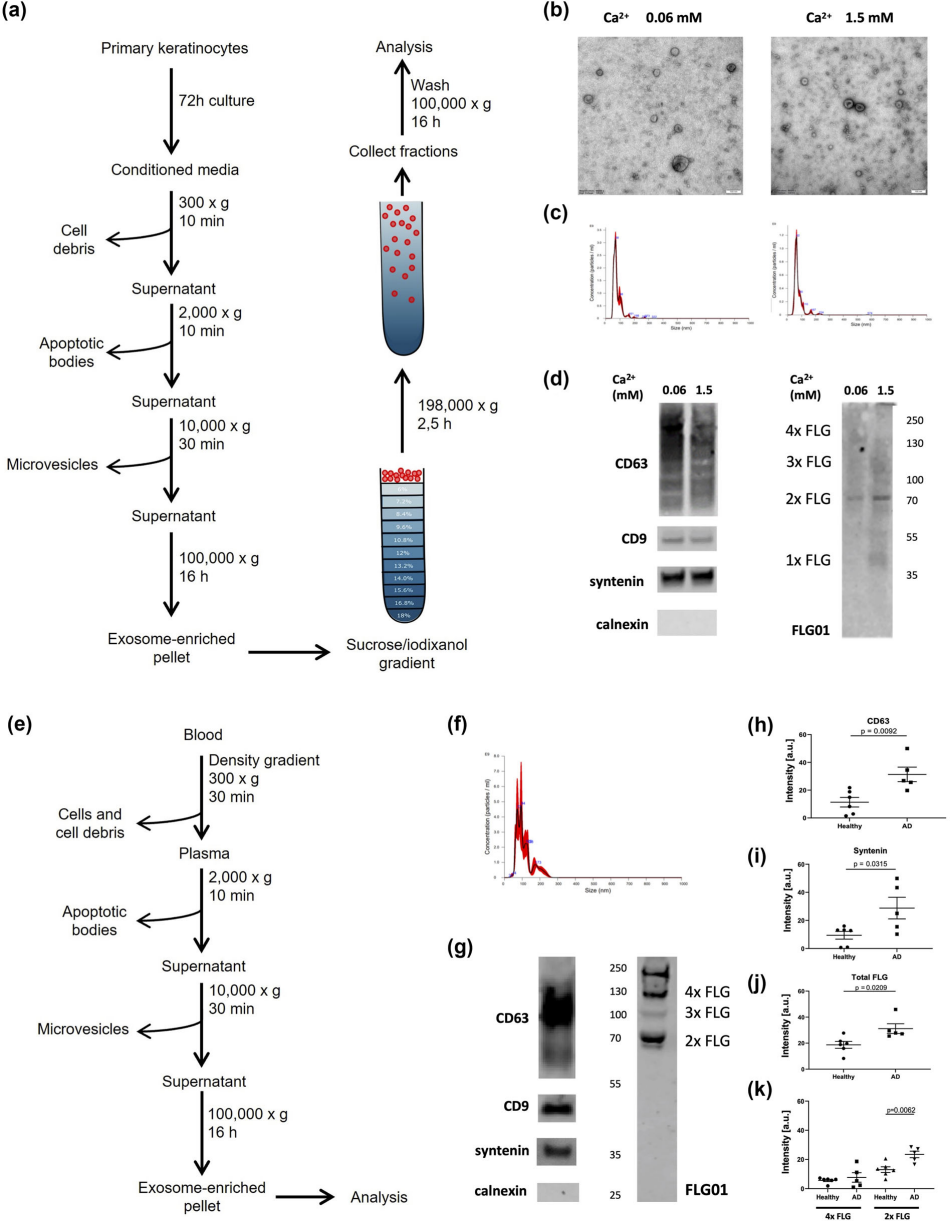
Profilaggrin/filaggrin is present in small extracellular vesicles secreted by keratinocytes and the blood of healthy individuals and atopic dermatitis patients. (a) Protocol for sEV isolation and purification from conditioned cell culture media; (b)–(c) confirmation of the typical sEV (b) morphology by electron microscopy and (c) size distribution by Nanoparticle Tracking Analysis of purified primary keratinocyte‐derived sEV/exosomes; (d) detection of exosomal markers and profilaggrin/filaggrin products in purified sEV/exosomes secreted by primary keratinocytes by western blot, example pictures shown (pooled fractions; *n* = 2); profilaggrin sequence features 10—12 filaggrin monomer repeats; the dimer consists of two such repeats joined by a linker; e) protocol for sEV isolation (100K pellet) from human blood plasma; (f) size distribution of human blood plasma sEVs (100K pellet) by Nanoparticle Tracking Analysis; *n* = 3; (g) Identification of exosomal markers and profilaggrin/filaggrin products in sEVs (100K pellet) isolated from human blood plasma, example pictures of *n* = 5 donors shown; (h)–(k) quantification of exosome marker and profilaggrin/filaggrin signal in western blot analysis of sEVs (100K pellet; sucrose/iodixanol‐purified) isolated from human blood plasma of healthy individuals and AD patients; data from *n* = 6 healthy donors and *n* = 5 AD patients, (h) CD63, (i) syntenin, (j) total filaggrin signal, (k) 2x filaggrin and 4x filaggrin; means ± SEM are shown, unpaired *t*‐test.

Next, we set out to confirm if profilaggrin/filaggrin can segregate as cargo in sEV secreted by keratinocytes. We cultured NHEKs and induced their differentiation using a standardised calcium switch assay (Figure S3). Small EV fractions (sEV), which contain both exosomes and smaller microvesicles (MVs) (Jeppesen et al., 2019) were then isolated from conditioned media by sequential centrifugation/ultracentrifugation method (exosome‐enriched 100K pellet, Figure 2a). We confirmed the characteristic ‘cup‐shaped’ morphology by electron microscopy as well as the expected size distribution by Nanoparticle Tracking Analysis (NTA) (Figure S4a–b and e–f). Since conditioned keratinocyte media could potentially contain KHGs released upon cell death and contaminate our 100K pellets, we next followed with purification and fractionation of those preparations by iodixanol/sucrose gradient (Figure 2a). Once again, electron microscopy and NTA analysis confirmed the expected morphology and size distribution of the density gradient‐purified sEVs (called ‘sEV/exosomes’ from now on) (Figure 2b–c). Western blot showed that top fractions 1–5 contained vesicles positive for exosomal markers (Figure S4c–d and g–h); we noted that syntenin expression was not well pronounced for some donors. Filaggrin‐related bands (incl. products of profilaggrin processing) were observed in both the sEV/exosomal fractions and in fractions containing small MVs (Figure S4d and h). Bands were also observed in the last iodixanol/sucrose gradient fraction (fraction 11), either due to the presence of KHGs or aggregated material released from ruptured sEV/exosomes; we noted that the signal was not detectable in some of the donors. Intriguingly, we did not observe any substantial differences in the filaggrin‐related cargo as far as the calcium level was concerned which is unexpected given the large increase in filaggrin expression upon NHEK differentiation. To improve the sensitivity of detection we next pooled fractions 1–5 (Figure 2d) adjusting the samples against cell counts (as in (Grisard et al., 2022; Tkach et al., 2022; Wahlund et al., 2017)), which revealed the band of ∼75 kDa in size (possibly filaggrin dimer, Figure 2d) to be the most prominent; the 37 kDa filaggrin monomer band could be also sometimes detected at lower levels in the vesicles harvested from differentiated NHEKs. Unexpectedly, the differences between sEV/exosomes obtained from the two calcium conditions were relatively small, suggesting that the excess is removed, above what is stored in KHGs, to maintain low level of the unsequestered protein.

Given our results suggesting that profilaggrin/filaggrin is removed from the cell, blocking of the sEV release should lead to the accumulation of potentially cytotoxic monomer and be detrimental to the cells. To verify this, we carried out experiments with exosome/sEV inhibitor GW4869 in immortalised primary keratinocyte line N/TERT‐1 (Dickson et al., 2000), within the course of 48 and 72 h. The treatment resulted in visible changes in the treated keratinocytes in comparison to the control at both time points, i.e., we observed increased vacuolisation and cell granularity, change of shape and reduced contact with the substratum (Figure S5). Interestingly, there was a difference between low and high calcium conditions, that is, the differentiated cells, subjected to GW4869 were more resistant and showed less pronounced effect, which could be potentially explained by their increased ability to store profilaggrin.

Taken together, we determined that filaggrin and profilaggrin‐processing products are sorted into keratinocyte‐derived sEV/exosomes in vitro, also in cells capable of proliferation, in which KHGs are not yet present and that preventing the sEV release is detrimental to the cells.

### 2.3 Profilaggrin/filaggrin cargo‐containing sEVs are found in the circulation

The size and flexibility of smaller EVs, especially exosomes, allow them to be carried into the circulation in vivo. Hence, we envisaged that we may be able to detect profilaggrin/filaggrin‐signal in blood derived samples; interestingly, filaggrin signal could be found in the total plasma as reported by Protein Atlas (Fig S6a; Peptide Atlas). To this end, since it is unlikely that our samples could be contaminated with KHGs, we used a simplified protocol (Figure 2e) to obtain exosome‐enriched sEV fractions from plasma of healthy donor samples; we identified a signal corresponding to the unique filaggrin‐relevant peptide present within repeat 1, 2, 6 and 10 by mass spec analysis (Figure S6b‐d), and verified that the vesicles of expected size (Figure 2f) contained profilaggrin/filaggrin‐relevant bands (Figure 2g). The signal was very strong and distinct bands corresponding to profilaggrin‐processing products could be easily identified but we also noticed that the 37 kDa band was not easily detectable in this case, suggesting potential degradation of the filaggrin monomer in the circulating sEVs. Subsequent gradient fractionation confirmed that filaggrin was present in the sEVs expressing exosomal markers (Figure S7a‐b). In contrast to the culture supernatants, the signals of the tetraspanins CD9 and CD63 was much lower in the fractions 6–10 and the signal for profilaggrin/filaggrin was not seen in those fractions, suggesting that small MVs bearing some of this cargo (as based on the results above) were not detected in the plasma 100K pellets.

### 2.4 Blood of AD patients contains exosomal filaggrin cargo

Decreased filaggrin expression resulting from a *FLG* mutation and/or downregulating effect of inflammatory mediators (Gutowska‐Owsiak et al., 2014, 2012, 2011; Howell et al., 2007) is a hallmark of AD skin. AD tissue sections demonstrate low KHG abundance; we hypothesized that this a direct consequence of low filaggrin production, not allowing to reach the KHG formation threshold and that this could support continuous cytoplasmic diffusion and redirection of unsequestered protein into the exosomal compartment to maintain cell‐tolerated level of IF binding. To investigate this, we first compared the exosome‐enriched sEV fractions isolated from equal plasma volumes obtained from healthy donors and AD individuals (who were not *FLG* mutation carriers), by assessment of the WB markers and determined that AD patients had an increase in the overall fraction of circulating sEVs/exosomes (Figure 2h‐i and S7c). Previously, a study reported an increase in the number of blood plasma sEVs/exosomes (100K pellet) in AD patients presenting with moderate‐to‐severe illness compared to healthy controls, but the difference was not statistically significant; however, the authors found the sEVs/exosomes of the AD patients to be slightly smaller (Chang et al., 2021). Next, we compared the size of the filaggrin‐related cargo; we noticed an overall increase of the combined 2x filaggrin and 4x filaggrin signal in AD plasma exosome‐enriched sEVs in comparison to those from the healthy controls (Figure 2j and S7d). The previously noted band at ca. 70 kDa was also more prominent in the patients, suggesting increased accumulation of the cleaved profilaggrin‐processing product corresponding to the filaggrin dimer (Figure 2k). Given the increased accumulation of sEV/exosomes in AD plasma, we also calculated the relative filaggrin load ‘per sEV/exosome’ and we found a non‐significant trend towards reduction in the AD patients (Figure S7e–j).

### 2.5 *S. aureus* exerts pronounced effect on the sEV compartment

**FIGURE 3:**
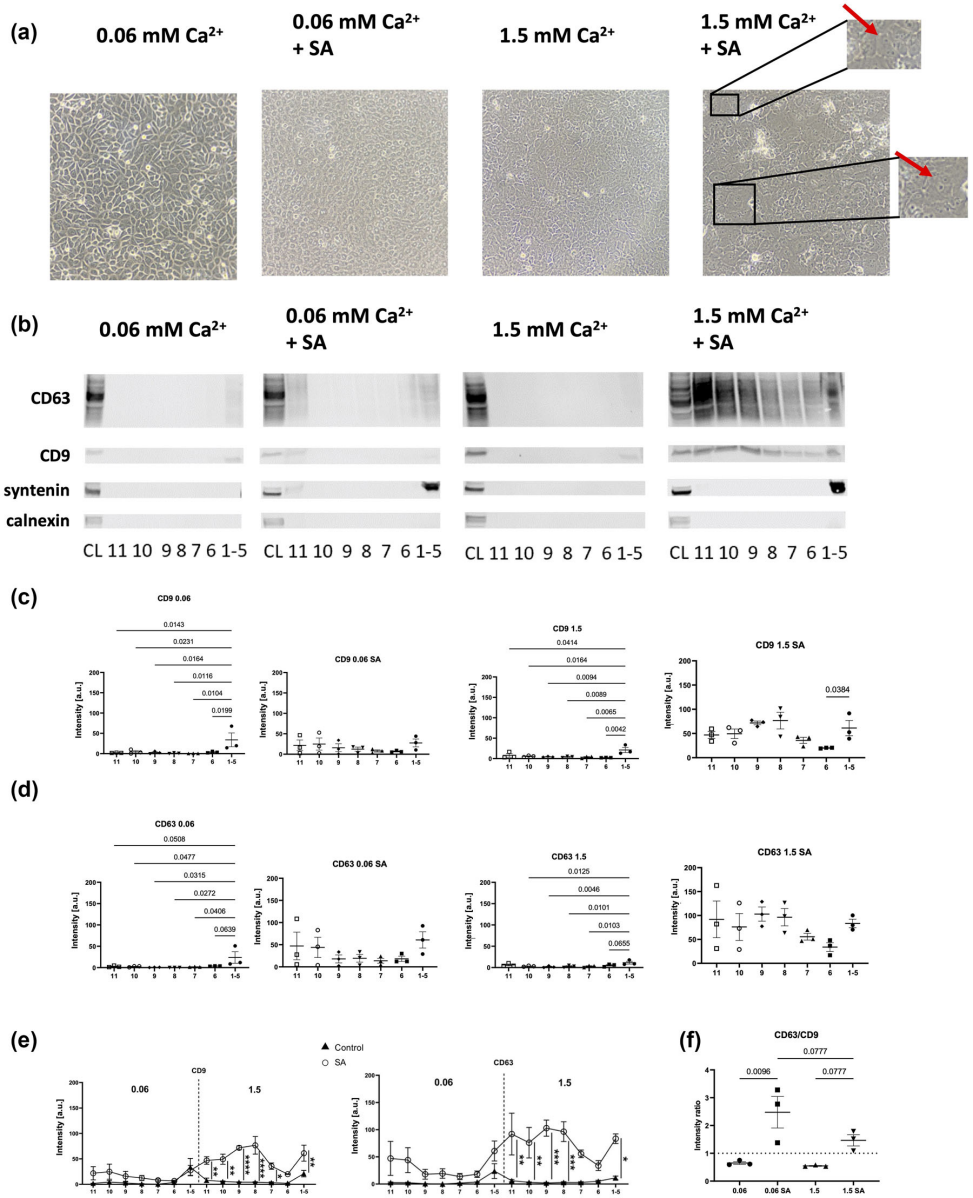
*S. aureus* dysregulates the small extracellular vesicle compartment in keratinocytes. (a) Example bright field microscopy pictures of N/TERT‐1 cells cultured at low or high calcium level and treated with *S. aureus* growth supernatant; *n* = 3 biological replicates; red arrows indicate syncytia; (b) detection of exosomal markers in iodixanol/sucrose gradient fractions following purification of sEV/exosomes produced by N/TERT‐1 keratinocytes treated with *S. aureus* growth supernatant by western blot, example data of *n* = 3 biological replicates shown; for 0.06 mM Ca^2+^ and 0.06 mM Ca^2+^ + SA cell lysates (CL) from the 0.06 mM Ca^2+^ untreated condition were used while for 1.5 mM Ca^2+^ and 1.5 mM Ca^2+^ + SA CL from the 1.5 mM Ca^2+^ untreated condition were used as controls; (c)–(d) quantification of the (c) CD9 and (d) CD63 signal in the post‐purification fractions; (e) comparison of CD9 and CD63 signal intensities measured in *S. aureus* growth supernatant‐treated versus untreated (control) conditions; *n* = 3 biological replicates; (f) CD63/CD9 signal intensity ratio comparison between conditions; quantified data are from *n* = 3 biological replicates, means ± SEM are shown, one‐way ANOVA with (c)–(d) Dunnett's correction, (e) Šidák's correction and (f) Holm‐Šidák's correction; SA, addition of *S. aureus* growth supernatant.

*S. aureus* is a common pathogen colonizing AD skin and contributing to the clinical deterioration in the patients. It has been shown that mildly acidic filaggrin breakdown products, that is urocanic acid (UCA) and pyrrolidone carboxylic acid (PCA) execute control over the bacteria in the skin (Miajlovic et al., 2010); reduced level of UCA and PCA in AD contribute to the elevated skin pH and loss of this antimicrobial control mechanism (Kezic et al., 2011). Exposure to the growth supernatant from the bacteria did not have any obvious effect on profilaggrin/filaggrin expression by NHEKs; however, we noticed increased processing of the protein, with increased presence of 37 kDa monomer band and breakdown products smaller in size (Figure S8a). This aligns with previous report suggesting that *S. aureus* promotes profilaggrin breakdown by increasing activity of endogenous serine proteases, such as kallikrein 5 (KLK5) in keratinocytes (Williams et al., 2017). Hence, we next investigated if the exposure of keratinocytes to *S. aureus* may influence the sEV/exosomal protein cargo. To this end, we exposed N/TERT‐1 grown at either low or high calcium level to *S. aureus* culture supernatant. We noticed extensive morphological changes in the treated cells, as expected given the *S. aureus* toxicity (Mempel et al., 2002), including formation of syncytia (red arrows) by the cells at high calcium level (Figure 3a). Strikingly, upon sucrose/iodixanol fractionation, we observed a pronounced dysregulation within the sEV compartment, as evidenced by increase of CD63 and CD9 signal in the fractions of the lower buoyancy in the gradient (fractions 6–11; Figure 3b–e). Interestingly, this was much more noticeable in the vesicles secreted by the calcium‐differentiated cells. Regrettably, due to the discontinuation of the previously used anti‐syntenin antibody a different reagent was used, which did not detect much signal in the control sEV fractions. However, very high syntenin presence in the exosomal fractions 1–5 could be still detected upon the *S. aureus* treatment, suggesting potential specificity for differentially modified (e.g., glycosylated) syntenin and suggesting induction of such form upon the treatment. Interestingly, CD63 was more liable for change in comparison to CD9 and we have observed an increase in the CD63/CD9 ratio also in the fractions 1–5 upon addition of the supernatants (Figure 3f). We showed that uninoculated *S. aureus* growth medium did not have an effect on the sEV compartment, confirming that it was the bacteria‐derived components present in the medium which were responsible its dysregulation (Figure S9a). Interestingly, secretion of MVs by N/TERT‐1 was also enhanced upon the treatment of these cells with *S. aureus* growth medium as evidenced by significant increase in CD9 signal and a non‐significant trend towards CD63 increase (Figure S9b).

### 2.6 S. aureus promotes filaggrin inclusion into the sEVs of the exosomal characteristics

**FIGURE 4:**
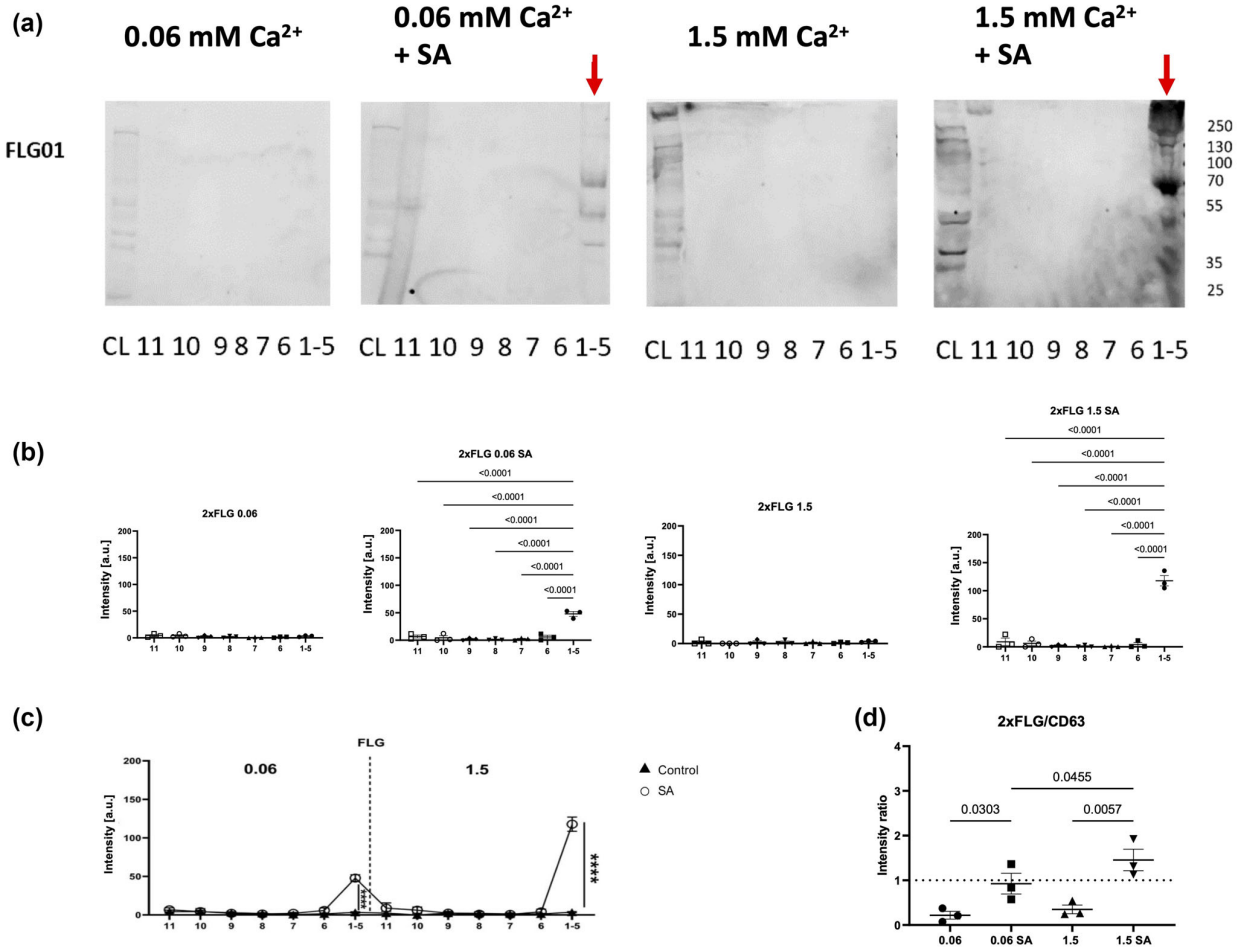
*S. aureus* promotes loading of profilaggrin/filaggrin into small extracellular vesicles. (a) Detection of profilaggrin/filaggrin products (red arrows) in iodixanol/sucrose gradient fractions following purification of sEV/exosomes produced by N/TERT‐1 keratinocytes treated with *S. aureus* growth supernatant by western blot, example data of *n* = 3 biological replicated shown; for 0.06 mM Ca^2+^ and 0.06 mM Ca^2+^ + SA cell lysates (CL) from the 0.06 mM Ca^2+^ untreated condition were used while for 1.5 mM Ca^2+^ and 1.5 mM Ca^2+^ + SA CL from the 1.5 mM Ca^2+^ untreated condition were used as controls; (b) quantification of the 2x filaggrin signal in the post‐purification fractions; (c) comparison of the 2x filaggrin signal in *S. aureus* growth supernatant‐treated versus untreated (control) conditions; (d) 2x filaggrin/CD63 signal intensity ratio compared between conditions; quantified data are from *n* = 3 biological replicates, means ± SEM are shown, one‐way ANOVA with (b) Dunnett's correction, (c) Šidák's correction and (d) Holm‐Šidák's correction; SA, addition of *S. aureus* growth supernatant.

N/TERT‐1‐secreted sEVs did not contain much profilaggrin/filaggrin cargo at baseline, possibly due to the individual characteristics of this particular keratinocyte donor; we noticed this for some donors in the study as mentioned earlier. However, upon the treatment with *S. aureus* substantial content of profilaggrin and profilaggrin breakdown products in sEVs was found. We noticed that this was limited to the exosome‐enriched fractions 1–5 at both low and high calcium conditions, with greater effect in the latter (Figure 4a–c; arrows). In addition, treated samples also demonstrated noticeable signal in the fraction 11, most likely due to pulling down protein aggregates that pelleted on the bottom of the gradient; this, again, was not observed in the control conditions. As for the fractions 1–5, preferential inclusion of the filaggrin dimer band was evident again; in contrast, no relevant profilaggrin/filaggrin cargo was found in the remaining sMV fractions (Figure 4a–c); we ruled out that the effect was caused by bacterial growth media (Figure S9a). We also tested APs and MV harvested within the isolation protocol and found no signal for filaggrin in those (Figure S9b). Furthermore, when we analysed the ratio between the intensity of the most prominent filaggrin band in the fractions 1–5 versus the respective CD63 intensity, we also noticed an enhancing effect of both the *S. aureus* supernatant treatment and the calcium level (Figure 4d), which resulted in increased filaggrin loading into vesicles.

### 2.7 Pathways including proteins of innate recognition of *S. aureus* intertwine with profilaggrin processing and MVB‐related vesicle formation

**FIGURE 5:**
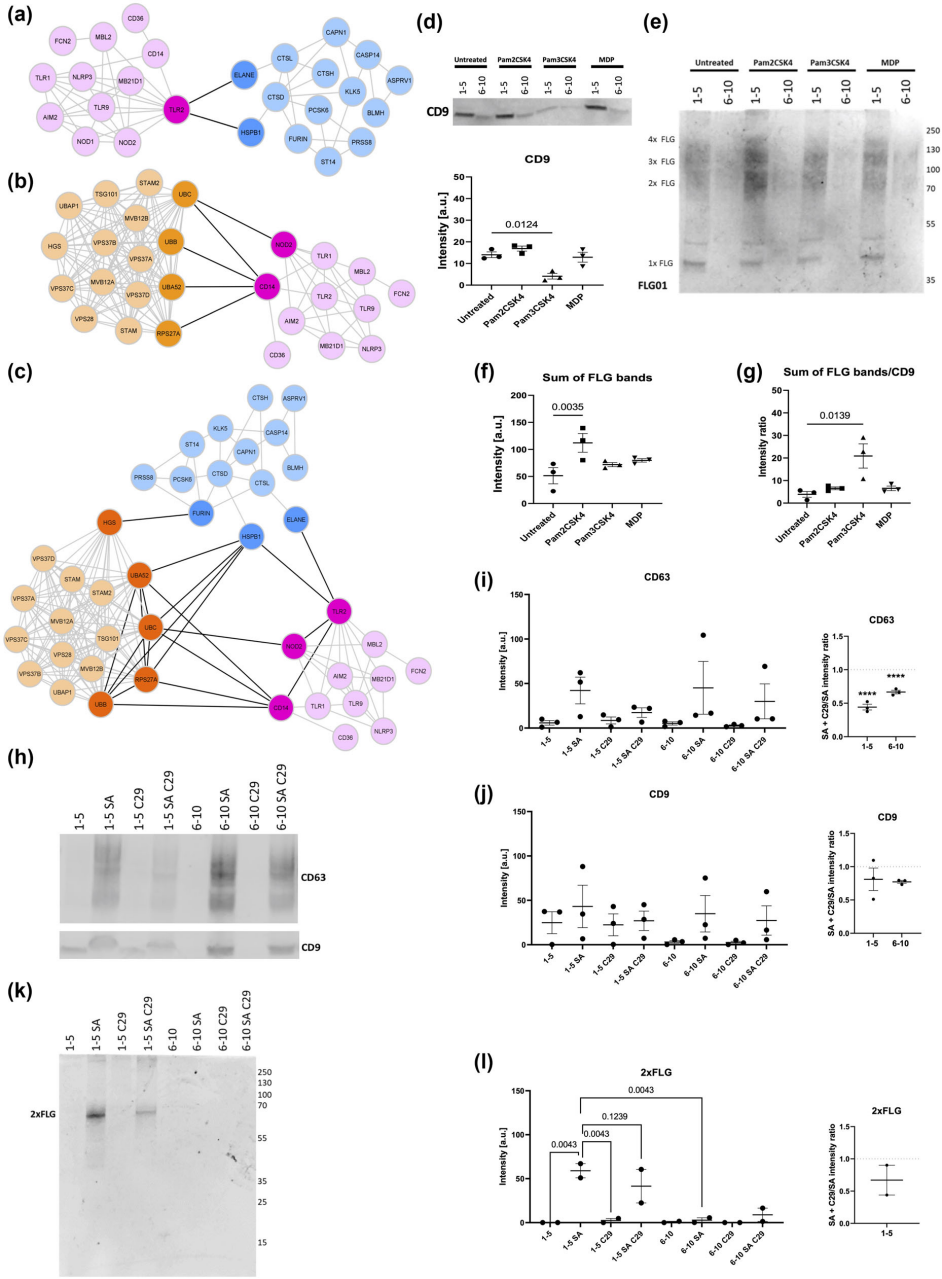
Loading of filaggrin into small extracellular vesicles is TLR2‐dependent. (a)–(c) STRING protein‐protein interaction map between *S. aureus*‐relevant pathogen recognition receptors (PRRs) and (a) profilaggrin‐processing enzymes, (b) proteins involved in sEV cargo sorting, (c) combination of the three networks; (d) presence of CD9 exosomal marker in pooled iodixanol/sucrose gradient fractions following purification of sEV/exosomes derived from N/TERT‐1 cells stimulated by TLR2 and NOD2 agonists by western blot and quantification of signals in pooled 1–5 fractions; *n* = 3 biological replicates; (e) profilaggrin/filaggrin signal in pooled iodixanol/sucrose gradient fractions following purification of sEV/exosomes derived from N/TERT‐1 cells stimulated by TLR2 and NOD2 agonists by western blot and (f) quantification of signals in pooled 1–5 fractions; (g) ratio between total filaggrin signal/CD9 signal ratio analysis in pooled 1–5 fractions; quantified data are from *n* = 3 biological replicates, means ± SEM are shown, one‐way ANOVA with (d) Šidák's correction and (e)–(g) Dunnett's correction; (h) detection of exosomal markers in pooled iodixanol/sucrose gradient 1–5 and 6–10 fractions following purification of sEV/exosomes derived from N/TERT‐1 cells treated with 100 μM C29, a TLR2 signalling inhibitor and stimulated with *S. aureus* growth medium by western blot; (i)–(j) quantification of (i) CD63 and (j) CD9 signal from (h); intensity ratios are calculated between signals from the *S. aureus* growth supernatant + C29 treatment/*S. aureus* growth supernatant‐only treatment conditions; *n* = 3 biological replicates; means ± SEM are shown, one‐way ANOVA with Holm‐Šidák's correction; ****p < 0.0001; (k) detection of profilaggrin/filaggrin in pooled iodixanol/sucrose gradient 1–5 and 6–10 fractions following purification of sEV/exosomes derived from N/TERT‐1 cells treated with 100 μM C29, a TLR2 signalling inhibitor and stimulated with *S. aureus* growth medium by western blot; (l) quantification 2xFLG signal from (k); intensity ratios are calculated between signals from the *S. aureus* growth supernatant + C29 treatment/*S. aureus* growth supernatant‐only treatment conditions; *n* = 2 biological replicates; means ± SEM are shown, one‐way ANOVA with Holm‐Šidák's correction; Pam2CSK4, TLR2/6 agonist; Pam3CSK4, TLR2/1 agonist; MDP, NOD2 agonist; C29, TLR2 signalling inhibitor.

Multiple pathogen recognition receptors (PRRs) are involved in the innate detection of bacterial pathogens in keratinocytes (Gutowska‐Owsiak & Ogg, 2012) and the epidermal tissue (De Koning et al., 2010). Hence, to understand the recognition route resulting in the observed increase in the profilaggrin/filaggrin sorting into exosome‐enriched sEVs, we next searched for recognised links between the innate pathways and profilaggrin‐processing enzymes using STRING pathway analysis (Szklarczyk et al., 2019). This analysis revealed very significant protein‐protein interaction score (PPI enrichment p‐value < 1.0e‐16) and highlighted node connections between two proteins implicated in profilaggrin/filaggrin processing (elastase and HspB1) and TLR2 receptor (Figure 5a). Interestingly, HspB1, which we have previously established as a critical protein implicated in the coordinated release of filaggrin from actin‐scaffold caged KHGs (Gutowska‐Owsiak et al., 2018), seems to constitute a bridge connection within the previously described TLR2‐KLK5 link (Yamasaki et al., 2011) (via cathepsin D). We next searched for connections between PRRs and the sEV‐sorting machinery, to determine if the innate recognition of *S. aureus* may have a potential to affect filaggrin sorting into the sEV/exosomal cargo. To this end, the analysis a list of 16 proteins identified by Reactome as those related to ‘cargo recognition and sorting’ (Table S1); yielding PPI enrichment p‐value < 1.0e‐16. The network identified NOD2 as potentially involved, as well as CD14, the known PRR co‐receptor for TLR4. Interestingly, those proteins also seem to show direct links to ubiquitin, that is UBA52, UBB, UBC and RPS27A (Figure 5b). Lastly, to increase the sensitivity of our search we proceeded with the analysis of the three combined protein networks (PPI enrichment p‐value < 1.0e‐16) which identified additional relationship between the clusters, that is a link between furin and hepatocyte growth factor‐regulated tyrosine kinase substrate (HGS) (Figure 5c). Strikingly, the search identified HspB1 as a central point where the three clusters converged.

### 2.8 Loading of the profilaggrin/filaggrin‐cargo into sEVs is TLR2‐dependent

To experimentally validate the results of our protein network analysis we next investigated changes in the regulation of the sEV compartment as well as the presence of profilaggrin/filaggrin cargo in sEVs upon stimulation of the cells with agonists of identified PRR (TLR2 and NOD2), using immortalized keratinocyte line (N/TERT‐1 (Dickson et al., 2000; Smits et al., 2017)). Here we focused on the cells cultured in the presence of 1.5 mM Ca^2+^ as the EV compartment dysregulation and the increase in filaggrin levels in sEVs upon the treatment of cells with *S. aureus* supernatant were much more pronounced in the differentiated cells. Since TLR2 is known to form functional heterodimers with either TLR1 or TLR6, we investigated the effects of the activation of both those heterodimers separately to obtain mechanistic information. With those agonists, we identified CD9 in fractions 1–5 only excluding involvement of those receptors in the previously observed EV compartment dysregulation induced by *S. aureus* (Figures 5d and S8b). However, we found a significant decrease in the intensity of the CD9 signal in fractions 1–5 upon stimulation by a TLR2/1 agonist, Pam3CSK4 (Figure 5d), suggesting a potential reduction of the exosomal output by this stimulation; however, we noticed no difference in the total profilaggrin/filaggrin‐relevant cargo (combined 4x filaggrin, 3x filaggrin and 2x filaggrin). Interestingly, however, there was a very pronounced enrichment of those profilaggrin cleavage products in fractions 1–5 signal from the filaggrin bands for N/TERT‐1 stimulation with a TLR2/6 agonist Pam2CSK4, (Figure 5e–f). Further calculation of the filaggrin/CD9 ratio (a proxy for cargo inclusion per vesicle) revealed that the engagement of the TLR2/1 heterodimer forced more efficient loading of the protein into the vesicles (Figure 5g). These results suggest that both TLR2/6 and TLR2/1 stimulation contribute to the changes in expulsion of filaggrin from the cells, by either increasing overall amount of the filaggrin cargo export or its loading efficiency under a condition of reduced exosomal output, respectively.

Since TLR2 seemed to be predominantly involved, we next followed with blocking experiments, using C29, a selective TLR2 inhibitor (Figure 5h–l). Here we observed that preincubation of N/TERT‐1 keratinocytes with C29 reduces the effect of *S. aureus* supernatant. Specifically, reduction of CD63 expression was observed in both exosome‐containing fractions 1–5 and fractions 6–10 in which predominantly sMVs were present (Figure 5i); this decrease was significant for ratios of CD63 signal from *S. aureus* supernatant + C29 versus *S. aureus* supernatant alone (Figure 5i), but it was not significant for CD9 (Figure 5j). TLR2 signalling blocking also resulted in a trend towards a decrease in filaggrin dimer signal (Figure 5k–l).

### 2.9 Products of KLK5‐dependent cleavage of profilaggrin are enriched in ubiquitination sites

**FIGURE 6:**
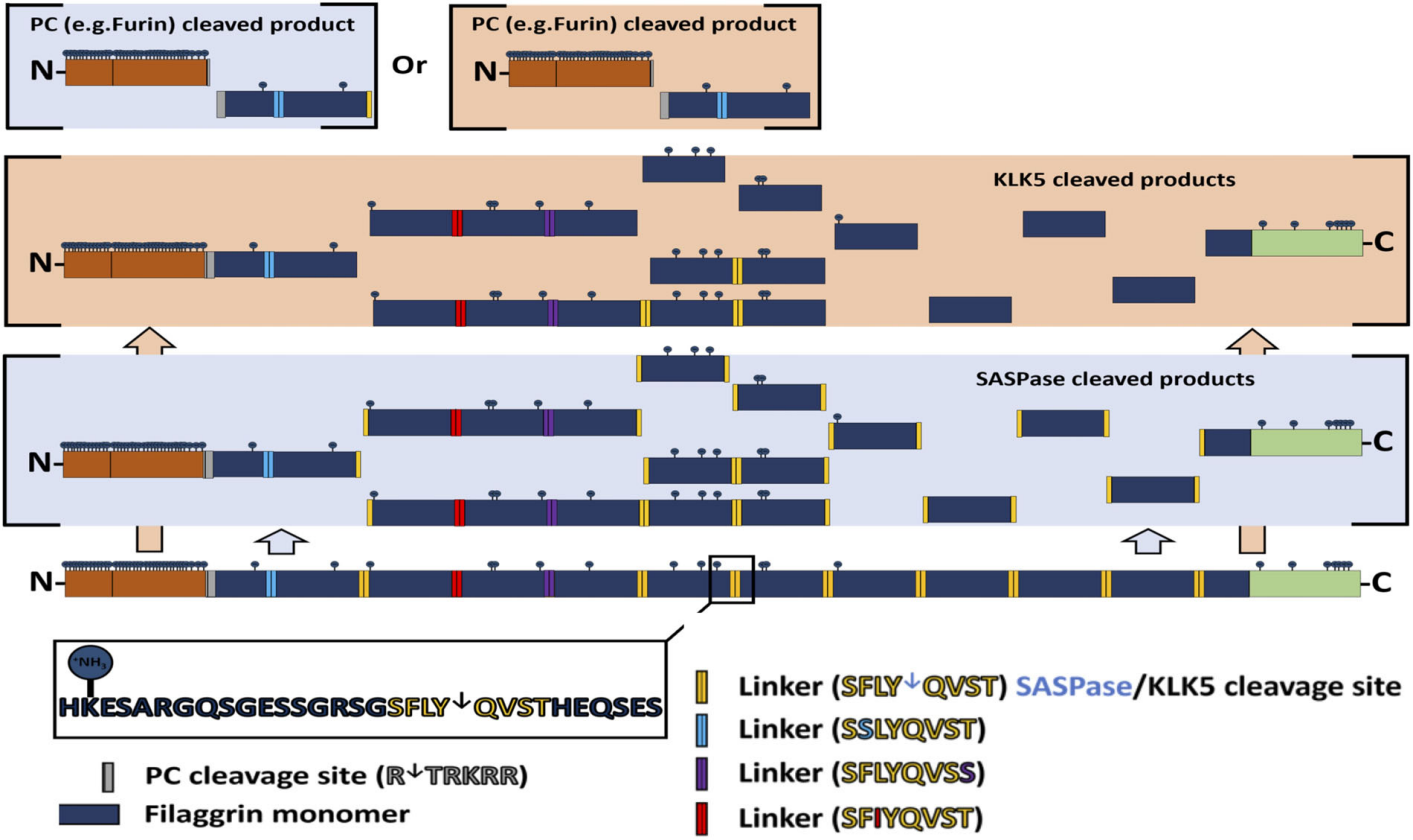
Cleavage products resulting from the activity of profilaggrin‐processing enzymes. Lys residues are indicated as potential ubiquitination targets.

Sorting of proteins into the cargo of intraluminal vesicles (ILVs) during the multivesicular body (MVB) formation has been shown to be frequently ubiquitin‐dependent (Ageta & Tsuchida, 2019). Hence, since the STRING analysis highlighted ubiquitination as a pathway of importance in the *S. aureus*‐exposed keratinocytes we asked if profilaggrin/filaggrin would be prone to such modification. This was especially interesting, since HspB1, which we identified as a central node between the three network clusters has been previously shown to bind ubiquitin and redirect ubiquitinated proteins (Parcellier et al., 2003); HGS, constituting a part of the ESCRT‐0 complex (Chou et al., 2015) has also been shown to recognise ubiquitinated substrates (Katz et al., 2002). Indeed, profilaggrin sequence contains multiple Lys residues (canonical ubiquitination marks) which could potentially undergo ubiquitination; interestingly, their spread is uneven within the profilaggrin sequence with the majority of Lys residues accumulated either within the N‐terminal domain or clustered at the C‐terminal end (Figure 6, Table S2). In contrast, filaggrin monomer repeats are much less Lys‐rich and only the first eight of those (including the 1^o^ partial repeat) contain at least one such amino acid (Figure 6, Table S2). Furthermore, when the KLK5 (Sakabe et al., 2013) and SASPase (Matsui et al., 2011) cleavage‐susceptible sites were considered, it appeared that many of the ∼37 kDa monomer‐sized products generated by the enzyme would contain no ubiquitination sites. In sharp contrast, all the larger products generated during the cleavage would contain several; these modelled cleavage products correspond to the bands observed by western blot. We also noted that the sixth linker region is flanked by five proximal sites of ubiquitin linkage (Figure 6), potentially masking the cleavage site. We modelled that in a such scenario, specific cleavage through linkers 5 and 7 would result in generation of a stable ∼70 kDa dimer product containing five ubiquitination sites. The high ratio between the number of ubiquitination sites and weight for this processing product (Table S2) could potentially explain the preferred inclusion of this product within luminal cargo which we observed.

## 3 DISCUSSION

Filaggrin is a protein with a plethora of biological roles supporting the epidermal barrier at both structural and immunological levels. This includes induction of programmed keratinocyte death (Presland et al., 2001) by free filaggrin monomers to assist *stratum corneum* formation; however, triggering this mechanism prematurely would likely result in a severe barrier defect. Our previous study on the mechanism controlling KHG‐dependent filaggrin sequestration (Gutowska‐Owsiak et al., 2018) determined that low numbers of small profilaggrin‐containing granules can be detected already in the lower suprabasal layers of the epidermis (Gutowska‐Owsiak et al., 2018). This implied that profilaggrin expression is not confined to the keratinocytes in the granular layer but likely initiated much earlier during the stratification process; however, we did not investigate cells with no visible granules in that study. Formation of KHG requires certain concentration threshold for either local precipitation (Gutowska‐Owsiak et al., 2018) or phase separation (Quiroz et al., 2020) to occur and filaggrin products of small size are not able to form granules, with a tendency to diffuse out into the cytosol (Quiroz et al., 2020). The actin cage‐involving, HspB1‐dependent mechanism that we previously defined relies on the profilaggrin accumulation supporting KHG formation and is unlikely to function until high protein production level is reached.

Here we confirmed that both the low cytoplasmic filaggrin expression and a degree of IF binding may be observed already in less differentiated cultured keratinocytes, as well as in the epidermal tissue, before the appearance of KHGs. There are serious implications to this; a complete lack of control over profilaggrin production and accumulation of (cytotoxic) monomers in the cell early during differentiation would be detrimental and result in cell death and stratification failure. Here, by integrating our data with results previously published by Presland et al. (2001) we propose that filaggrin expression and cellular localisation undergo sequential changes. Specifically, it begins with free diffusion of the nascent profilaggrin into the cytoplasm at early stages of keratinocyte differentiation, processing by profilaggrin processing enzymes i.e., KLK5 (Williams et al., 2017), furin (Pearton et al., 2001; Spencer et al., 2008), caspase‐14 (Kuechle et al., 2001), matriptase (Alef et al., 2009), prostasin (Alef et al., 2009), PACE4 (Pearton et al., 2001), BLMH (Gutowska‐Owsiak et al., 2014), which have been shown to be expressed at low levels in not yet fully differentiated cells as shown in several publications and indicated by data from ProteinAtlas; Table S3) and a certain tolerated level of protein binding to IFs. The mechanism allows for activation of the positive feedback‐driven intensification of filaggrin expression. This diffusion decreases, however, when the local concentration reaches the threshold needed for KHG formation. Such dynamics must be accompanied by an active controlling mechanism to maintain ‘safe’ intracellular filaggrin level that the cell can survive, below a certain ‘death threshold’ as previously showed by regulated filaggrin overexpression experiments carried out by Presland et al. (2001). In the current study we show that profilaggrin/filaggrin‐derived products are efficiently removed from the keratinocyte cytosol by the means of sEV/exosomal export; blocking of this process by GW4869 inhibitor had cytotoxic effects which mirrors the effect of forced filaggrin expression by transfection. We propose that this mechanism enables the cells to maintain safe cytoplasmic levels of filaggrin monomers during the process of profilaggrin synthesis intensification to allow for KHG formation. In agreement with this, our data show that inhibition of release of exosomes/sEVs is detrimental to keratinocytes and that cells capable of forming KHGs are more resistant to this treatment. We believe that this novel and basic homeostatic mechanism permits progression through stratification, essential during the process of the epidermal barrier formation and would be in line with a cell type‐specific function; utilising the sEV system, already present in keratinocytes for this purpose would be, however, beneficial beyond the cellular or tissue level.

Our study also indicates that filaggrin‐containing exosomes are transferred into the bloodstream, which may have broader implications systemically, important for the protein of very confined expression pattern, mainly restricted to the epidermis. Interestingly, while the relative abundance of profilaggrin/filaggrin cargo ‘per a vesicle’ remains relatively unchanged, the total detected signal is increased in exosomes isolated from AD plasma, due to the generally increased abundance of sEV/exosomes in the blood of the patients. Consistent with this, recent studies identified increased filaggrin levels in total serum samples from patients with different allergic manifestations (Bin Saif et al., 2018; Rasheed et al., 2018). We hypothesize that the reduced cellular profilaggrin expression in AD, set at the level insufficient to support KHG formation forces re‐direction of the nascent protein into the exosomal secretory pathway and removal from keratinocytes to avoid cell death; these sEV/exosomes can be found in the circulation. While this mechanism could be important at the level of cellular homeostasis, it could further compound barrier dysfunction in the disease due to increased depletion of filaggrin from the epidermis. Of note, given the strong signal observed in the exosomes/sEVs isolated from the blood, inclusion of profilaggrin‐related products inside those vesicles seems to provide protection from the proteases in the extracellular space and the bloodstream; this ensures efficient long‐distance filaggrin transport into distant tissues.

Currently, we do not know the consequences of the profilaggrin/filaggrin cargo presence in the circulation and transfer to peripheral tissues in the healthy individuals or AD patients. However, filaggrin has a documented suppressive effect on innate immunity pathways as well as antigen‐specific T cell responses. Specifically, we have previously documented that filaggrin‐insufficient keratinocytes induce higher reactivity of antigen experienced T cells to presented peptide antigens (Marwah et al., 2014); the exact mechanism is unknown. As for the lipid‐specific T cells, filaggrin has been documented to exert direct inhibitory action on the lipid neoantigen‐generating phospholipase A2 (PLA2) enzyme (Jarrett et al., 2016). Adding to that, we have very recently determined that sEV membranes themselves contain lipid antigens suitable for CD1a presentation and are capable of inducing protective and homeostatic T cell responses (Kobiela et al., 2022). However, filaggrin insufficient keratinocytes secrete sEVs characterised by altered lipidome which results in a bias of the CD1a‐dependent T cell responses from IFNγ‐dependent into type 2 inflammation with increased IL‐13 secretion, thus contributing to allergic inflammation. Currently we do not know which cells could receive this systemically distributed cargo; following intravenous injection, vesicles of the sEV size predominantly accumulate in the liver, kidney and spleen (Sun et al., 2010; Zhang et al., 2020); these tissues are not the main sites of inflammation in allergic march. However, some studies also found sEV accumulation in the lungs (Sun et al., 2010); it remains to be tested in the animal models if the sEV‐mediated filaggrin delivery to distant organs in AD may prevent allergic inflammation, via the impact on either innate or adaptive immune responses and alter the progression of the allergic march.

Increased level of vesicular secretion has been observed in conditions leading to cell death (Baxter et al., 2019) and this may include enhanced level of blebbing from the plasma membrane and generation of, so called, ‘apoptotic exosome‐like vesicles’ (AEVs) (Kakarla et al., 2020); such AEVs have been shown in pathology as a major contributor to inflammation, stimulating expression of proinflammatory mediators and acting as damage‐associated patterns (DAMPs) (Park et al., 2018). In the second part of the study, we show that *S. aureus*, a pathogen successfully spreading in the skin of AD patients (Totté et al., 2016) and contributing to clinical exacerbations may disturb the sEV/exosomal system in keratinocytes, possibly via this mechanism, but with an additional effect on profilaggrin/filaggrin cargo sorting. It is known that *S. aureus* actively targets pathways of keratinocyte differentiation and epidermal barrier formation (Hirasawa et al., 2010), including expression of filaggrin itself (Son et al., 2014) and profilaggrin processing enzymes, i.e calpain‐1, caspase‐14 and kallikrein‐5 (Son et al., 2014; Williams et al., 2017). *S. aureus* seems to be under control in healthy skin partly thanks to the acidic pH of the skin surface supported by the accumulation of small filaggrin‐breakdown products of the natural moisturising factor (NMF), i.e., UCA and PCA, which control bacterial growth (Miajlovic et al., 2010). Rapid filaggrin release from keratinocytes dying upon the exposure to bacterial toxins, leading to the intensified NMF generation in the epidermis, would therefore be detrimental to the pathogen. Our study proposes that the bacteria may repurpose the basic homeostatic mechanism contributing to the formation of a protective epidermal barrier that we discovered to reduce the level of available filaggrin in the skin. Specifically, stimulation of profilaggrin cleavage coupled with the cellular redirection and loading into the exosome‐enriched sEVs, which are naturally flushed away into the circulation, would result in enhanced filaggrin expulsion from the skin. Such intensified filaggrin removal process could support bacterial growth and colonisation and contribute to the increased amount of filaggrin‐related sEV cargo in the circulation of AD patients. It would be interesting to investigate the secretion of profilaggrin/filaggrin cargo within sEVs in real time, also in patients heavily colonised with *S. aureus*, for example, by open flow microperfusion on human skin, which would provide additional in vivo data; this unfortunately was not possible in the current study. Recently, we demonstrated that the exposure of keratinocytes to *Candida albicans* in the context of AD inflammatory *milieu* leads to re‐programming of the surface glycosylation pattern on secreted sEVs; sEVs released under these conditions increased their capacity to interact with dendritic cells (DCs) via inhibitory Siglec receptors (Kobiela et al., 2022), activation of which has been shown to reduce innate responses to pathogens (Alef et al., 2009; Rasheed et al., 2018). To our knowledge, no extracellular vesicle‐assisted mechanism to reduce host control measures by removal of proteins or compounds with antimicrobial properties from the cells or tissue has been described to date; whether this is a broadly pathogen‐utilised mechanism or *S. aureus*‐specific phenomenon remains to be clarified. However, filaggrin was previously detected in the unfractionated serum samples from patients with sepsis caused by several different bacteria species (Garcia‐Obregon et al., 2018); while the authors interpreted this finding as sample contamination during handling it is plausible that sEVs were the source of the protein.

On the other hand, changes in vesicle secretion upon the exposure to toxins and bacterial cellular constituents could also represent a mechanism utilized by keratinocytes as a part of pathogen defence to initiate immune cell infiltration. Small extracellular vesicles, and especially exosomes, including those secreted by non‐immune cells (Hovhannisyan et al., 2021), have been shown as important participants in both innate and adaptive immune responses (Schorey et al., 2015; Wahlund et al., 2017). With their small size and high durability, sEVs are particularly suitable for long‐distance intercellular communication, including within the immune system. In the skin this could constitute a route allowing for alarming of sentinels, i.e. Langerhans cells (LCs) in the epidermis, or dermal DCs. To this end, secretion of sEVs in the context of infection and cell death could result in enhanced transfer of DAMPs to those cells. It is not clear how filaggrin transfer within/out of the skin could benefit the host; however, given that the protein has immunoregulatory function, e.g., as an inhibitor of PLA2 (Jarrett et al., 2016) in the CD1a‐mediated antigen presentation pathway, and as a suppressor of LC activation (Leitch et al., 2016); hence expulsion of filaggrin away from the site of inflammation would likely promote pathogen clearance.

We determined that the process of enhanced filaggrin loading into sEV/exosomes results from the TLR2 signalling intertwined with the enzymatic processing and MVB sorting networks^3941^. TLR2 is the primary receptor for innate recognition of gram‐positive bacteria, including *S. aureus* (Takeuchi et al., 1999), with an important role during CD1a‐mediated T cell responses (Hardman et al., 2017); its polymorphism has been shown in AD previously (Tesse et al., 2011) and linked with the disease severity as well as other allergic manifestations (Potaczek et al., 2011). Our modelling determined that PRR and vesicle networks converge at the level of HspB1 and HSG, which seem to link the exposure to *S. aureus* with the ubiquitination process (Katz et al., 2002; Parcellier et al., 2003). On this note, we have recently found that profilaggrin undergoes ubiquitin‐proteasome system (UPS)‐mediated turnover, with relevance to the disease, especially with the context of *FLG* mutations, which alter predicted stability of this protein (Paul et al., 2023). Given the enrichment in the ubiquitination marks, it is hence plausible that ubiquitination could mediate profilaggrin/filaggrin trafficking to the endocytic system (Ageta & Tsuchida, 2019) and sorting into exosomes at the level of MVBs, beyond its role in proteasomal degradation (Liao et al., 2022); it could also participate in gene regulation by promoting nuclear localisation (Liao et al., 2022). Hence, any affect that *S. aureus* has on this system, e.g., by enhancing profilaggrin cleavage and generation ubiquitinated products may be important and potentially, targeted therapeutically.

Taken together, we propose that sEV/exosome‐aided removal of profilaggrin/filaggrin products exists in keratinocytes to regulate intracellular free filaggrin monomer content and prevent premature cell death during stratification. We believe that this system is of the greatest importance early in epidermal differentiation, before the appearance of KHGs, when it is replaced by the AKT1‐HspB1‐actin cage‐dependent mechanism (Gutowska‐Owsiak et al., 2018). While it is conceivable that this mechanism could have additional roles, e.g., constitute a part of keratinocyte pathogen defence system, enhanced filaggrin expulsion from the skin promoted by *S. aureus* inducing profilaggrin processing and MVB sorting into sEVs may benefit the bacteria by reducing the impact of antimicrobial host control and safeguarding its growth. Our study suggests that this pathologic mechanism could be explored as a novel target for targeted therapeutic interventions.

## 4 METHODS

### 4.1 Skin and blood samples

Skin samples were collected from healthy donors undergoing surgery on the ethical approvals from UK National Research Ethics Service (14.NW.1153) and the Independent Bioethics Committee for Scientific Research at Medical University of Gdansk (ethical approval numbers: NKBBN/559/2017‐2018, NKBBN/621‐574/2020 and NKBBN/746/2019‐2020) into PBS (Sigma‐Aldrich, St. Louis, MO, USA), with 1% penicillin and streptomycin (Sigma‐Aldrich, St. Louis, MO, USA). Peripheral blood samples were used with the ethical permission NKBBN/621/2019 and the Independent Bioethics Committee for Scientific Research at Medical University of Gdansk. The patients in our study were tested for the *FLG* mutations present in the Polish population (rs2282del4, R501X, R2447X and S3247X) and were all found to be wild type.

### 4.2 Keratinocyte isolation and culture

Skin samples were stored in cold PBS with the addition of 100 U/ml penicillin and 100 μg/ml streptomycin (Sigma‐Aldrich, St. Louis, MO, USA). For cell isolation subcutaneous adipose tissue was removed before incubation in dispase (12 U/ml, Corning, NY, USA). Epidermal sheets were harvested and digested at 37°C and 0.25% trypsin‐EDTA solution (Sigma‐Aldrich, St. Louis, MO, USA). Keratinocytes were seeded on a collagen IV‐coated dishes (Corning, NY, USA) and cultured in EpiLife medium (with the addition of EDGS, and antibiotics as above and 10% FBS). The following day, the medium was replaced with a serum‐free EpiLife (with EDGS and antibiotics) and cells were cultured at 37°C, 5% CO_2_. NHEKs were cultured at 37°C, 5% CO_2_, in animal product‐free EpiLife medium (Thermo Fisher Scientific, Waltham, MA, USA) with low calcium (0.06 mM) and EpiLife™ Defined Growth Supplement (EDGS; Thermo Fisher Scientific, Waltham, MA, USA) with addition of 100 U/ml penicillin + 100 μg/ml streptomycin (Sigma‐Aldrich, St. Louis, MO, USA). Pooled NHEK cultures from *n* = 3‐4 donors were used when necessary to obtain enough sEVs (harvesting from 2D culture experiments). N/TERT‐1 immortalised keratinocyte cell line (Dickson et al., 2000; Smits et al., 2017), a kind gift from Prof Rheinwald was cultured in keratinocyte serum‐free medium (K‐SFM) (Thermo Fisher Scientific, Waltham, MA, USA) supplemented with 25 μg/ml bovine pituitary extract (BPE) (Thermo Fisher Scientific, Waltham, MA, USA), 0.2 ng/ml epidermal growth factor (EGF) (Thermo Fisher Scientific, Waltham, MA, USA), 100 U/ml penicillin + 100 μg/ml streptomycin (Sigma‐Aldrich, St. Louis, MO, USA) and 0.4 mM Ca^2+^ at 37°C, 5% CO_2_. For maintenance of the cell line cells were subcultured at no more than 35% confluence. Serum‐free EpiLife medium supplemented as described above was used as ‘EV‐free medium’ for N/TERT‐1 cells. Cell treatments and EV‐free media switches were carried out when the cells reached 80%–90% confluence.

### 4.3 Epidermal sheet isolation, antibody staining and image analysis

Epidermal sheets were isolated by incubation in dispase overnight (5 U/ml; Sigma‐Aldrich, Gillingham, Dorset, UK) and epidermis was separated from dermal tissues manually with forceps. The sheets were then incubated in 4% formaldehyde (Sigma‐Aldrich, Gillingham, Dorset, UK), and 0.1% Triton X‐100 (Sigma‐Aldrich, Gillingham, Dorset, UK) and then blocked in the buffer (5% FCS, Sigma‐Aldrich, Gillingham, Dorset, UK; 2% BSA, Sigma‐Aldrich, Gillingham, Dorset, UK in PBS; or 0.4% fish skin gelatin dissolved in TBS and 0.2% Triton X‐100). Anti‐filaggrin G‐20 antibody (Santa Cruz Biotechnology, Dallas, TX, USA) was used followed with the secondary anti‐goat Alexa 488 (Life Technologies, Waltham, MA, USA). Nuclei were visualized by Hoechst (NucBlue, Life Technologies/Thermo Fisher Scientific, Waltham, MA, USA). The epidermal sheets were mounted on microscope slides and cover‐slipped with Mowiol‐488 (Sigma‐Aldrich, Gillingham, Dorset, UK). Data acquisition was carried out on the Zeiss 780, Zeiss LSM 710 inverted confocal microscope (Zeiss, Jena, Germany) by recording 2D images in different axial (3D) planes. Then, every single confocal plane of a 3D imaging stack was deconvolved using the Huygens Professional software package (Huygens; Scientific Volume Imaging, Hilversum, the Netherlands). Deconvolution allows minimizing image artifacts due to noise and optical aberrations. A theoretical point‐spread function (PSF) defined by the software (adjusted as a function of the penetration height/depth) was employed for deconvolution of the 3D images and when 2D planes were used, we employed both our defined PSF (as in (Gutowska‐Owsiak et al., 2018)) and the theoretical one developed by the software (noting no significant difference). The resulting 3D image was surface rendered (Huygens) to easily observe granules and nuclei without a large contribution from the background and minimizing the effect of in‐depth aberration imaging. Raw data was also presented as in (Supplementary data) to show the level of background signal coming from the filaggrin at different depths. Selected planes along the z‐axis were shown to highlight the localization and distribution at single planes of the filaggrin.

### 4.4 EV isolation

EV isolation was carried out from EV‐free media after 72 h of culturing. Briefly, conditioned medium (CM) was centrifuged at 300 x g (Megafuge 16R TX‐400 centrifuge, Thermo Scientific, Waltham, MA, USA) for 10 min at 4°C to remove the cells and cell debris, followed by a spin at 2000 x *g* (Megafuge 16R TX‐400 centrifuge, Thermo Scientific, Waltham, MA, USA) for 10 min at 4°C to remove soluble proteins and apoptotic bodies (AP). The supernatant was ultracentrifuged (OptimaTM L‐90K or OptimaTM LE‐80K ultracentrifuge, Beckman Coulter, Brea, CA, USA) at 10,000 x *g* for 30 min at 4°C to isolate the pellet of microvesicles (MVs) which was washed and stored as above. The supernatant was ultracentrifuged at 100,000 x *g* for 16 h at 4°C to pellet exosome‐enriched small extracellular vesicle fraction (sEV; 100K pellet). The sEV pellet was washed in PBS as above and stored at −80°C for further use. sEV pellet was purified using iodixanol/sucrose discontinuous gradient with iodixanol (OptiPrep™; STEMCELL Technologies, Vancouver, BC, Canada) concentration ranging between 6–18% (increments of 1.2%, 1 ml each fraction). 100K pellet was top loaded on the OptiPrep layers and ultracentrifuged at 198,000 x g for 2.5 h. Fractions were collected separately (1 ml), or pooled where indicated. The top‐loaded sample was pooled with the first OptiPrep fraction and together considered the first fraction. After that samples were ultracentrifuged at 100,000 x *g* for 16 h in PBS to wash the sEVs. Blood plasma samples were obtained by gradient centrifugation (Lymphoprep; STEMCELL Technologies, Vancouver, BC, Canada) over 20 min at 750 x *g* (Megafuge 16R TX‐400 centrifuge, Thermo Scientific, Waltham, MA, USA) with the centrifuge brake switched off. Collected plasma was diluted in PBS and EV isolation was carried out as above, starting from the 2000 x *g* spin.

Clinical samples were obtained from healthy donors and AD patients into vacutainers (containing EDTA, BD, Franklin Lakes, NJ, USA) and plasma was isolated by centrifugation over 10 min at 3000 x *g* (Eppendorf, Hamburg, Germany). Collected plasma was kept at −20°C for further use.

### 4.5 EV characterisation

For TEM visualization, the EVs were adsorbed onto formvar/carbon‐coated copper grids size 300 mesh (EM Resolutions, Sheffield, UK), then stained with 1.5% uranyl acetate (BD Chemicals Ltd.), and imaged by Tecnai electron microscope (Tecnai Spirit BioTWIN, FEI, Hillsboro, OR, USA). For the Nanoparticle Tracking Analysis (NTA) the EV samples were diluted 1000x in PBS and run using NS300 NanoSight NTA (Malvern Panalytical, Malvern, UK).

### 4.6 Western blot

1 × 10^6^ cells were lysed in 100 μl RIPA buffer (Cell Signalling Technology, Danvers, MA, USA) containing protease inhibitors (cOmplete™, Mini, EDTA‐free Protease Inhibitor Cocktail, Sigma‐Aldrich, St. Louis, MO, USA) and spun for 15 min at 4°C, 13,000 x *g*; the supernatant was harvested. 4x Bolt™ LDS Sample Buffer (Thermo Fisher Scientific, Waltham, MA, USA) (10x diluted) was added to the lysates or EV samples and the samples were heated for 10 min at 80°C. Samples were run on Bolt™ 4%–12% Bis‐Tris Plus Gels (Thermo Fisher Scientific, Waltham, MA, USA) for 30 min using PowerEase™ 300 W Power Supply (Thermo Fisher Scientific, Waltham, MA, USA). The proteins were transferred onto nitrocellulose membranes (iBlot™ 2 Transfer Stacks, nitrocellulose, regular size, Thermo Fisher Scientific, Waltham, MA, USA) using iBlot transfer system (iBlot 2 Dry Blotting System, Thermo Fisher Scientific, Waltham, MA, USA) and the membranes were blocked in 5% fat‐removed milk in PBS for one hour. Primary antibody incubations (diluted 1:100–1:1000) were carried out at 4°C on shaker overnight and secondary antibody IRDye® 800CW or IRDye® 680RD (LI‐COR Biosciences, Lincoln, NE, USA) (dilution 1:25,000) for 30 min at RT. The membranes were scanned and analysed using Odyssey Clx Imaging System (LI‐COR Biosciences, Lincoln, NE, USA). Signal intensity was quantified using ImageJ/Fiji 1.53f51 by the measurement of ‘mean grey value’ of the bands and background subtraction.

### 4.7 Cell treatments

Overnight culture medium of *S. aureus* ‘Newman’ strain (2.4 × 10^9^ CFU/ml) or uninoculated growth Brain Heart Infusion medium (BHI medium; Becton Dickinson, Franklin Lakes, NJ, USA) were spun at 1700 x *g*, 5 min. The supernatant was sterile‐filtered through a 0.1 μm pore size filter and stored at −20°C. N/TERT‐1 cells were grown to 80% confluence and treated with the *S. aureus* supernatant at the time of EV‐depleted media switch at 5% v/v of the total media. sEVs were isolated and density gradient‐purified as described above. sEVs isolated from conditioned media volume equivalent to 1.5 x T150 cell culture flasks (*S. aureus* supernatant) or 3 x T150 cell culture flasks (uninoculated growth medium) were loaded per well in SDS‐PAGE gel. For TLR2 signalling blocking experiments, N/TERT‐1 were preincubated with 100 μM of C29 (MedChemExpress, Monmouth Junction, NJ, USA) for 1 h before addition of the *S. aureus* supernatant.

For the PRR agonist treatment N/TERT‐1 cells were stimulated with 50 ng/ml of Pam2CSK4, 1 μg/ml of Pam3CSK4 or 10 μg/ml of MDP (all from InvivoGen, San Diego, CA, USA) at the time of EV‐depleted media switch. EVs were isolated and density‐gradient purified as described above. sEVs isolated from conditioned media volume equivalent to 3 x T150 cell culture flasks were loaded per gel well in SDS‐PAGE. For exosome release inhibition experiments N/TERT‐1 were grown to 80% confluence and incubated with 10 μM of GW4869 (MedChemExpress, Monmouth Junction, NJ, USA) in EpiLife medium supplemented as described above for 48 or 72 h.

### 4.8 Immunofluorescence

Eight‐well chamber slides (VWR International, Radnor, PA, USA) were coated with coating matrix (Thermo Fisher Scientific, Waltham, MA, USA), then NHEKs were seeded (50,000‐ 100,000 cells/well) in 500 μl cell culture medium. For staining the cells were fixed with 100 μl/well 4% formaldehyde in PBS for 5 min, then washed and permeabilized with 0.1% Triton x‐100, 100 μl per well, for 5 min. For blocking the permeabilized cells were incubated for 1 h with 5% FBS, 2% BSA in PBS on shaker. After blocking 200 μl of the primary Abs were added (1:100 in PBS) and incubated at RT 1 h with gentle shaking. NucBlue (Thermo Fisher Scientific, Waltham, MA, USA) was diluted in PBS (2 drops per ml) and the secondary Abs were diluted in NucBlue/PBS at 1:200. 200 μl of the diluted secondary antibodies were added and incubated 30 min at RT with gentle shaking. Samples were coverslipped with ProLong™ Gold Antifade Mountant (Thermo Fisher Scientific, Waltham, MA, USA) and images acquired on the Leica TCS SP8 x microscope (Leica Microsystems, Wetzlar, Germany).

### 4.9 Mass spectrometry

Small extracellular vesicles isolated from pooled plasma samples were subjected to lysis with 1% SDS and cysteine residues’ reduction by dithiothreitol and processed by the standard Multi‐Enzyme Digestion Filter Aided Sample Preparation (MED‐FASP) protocol (Wiśniewski & Rakus, 2014) with alkylation of cysteine residues by iodoacetamide and consecutive digestion by LysC, trypsin, and chymotrypsin. Peptide fractions resulting from each digestion step were collected, desalted in a standard STAGE Tips procedure (Rappsilber et al., 2007), and analyzed in the data‐dependent acquisition mode on a Triple TOF 5600+ mass spectrometer (SCIEx, Farmingham, MA, USA) coupled with an Ekspert MicroLC 200 Plus System (Eksigent Technologies, Redwood City, CA, USA). Resulting files were analyzed in a single database search by PEAKS Studio 10 (Bioinformatics Solutions Inc., Waterloo, ON, Canada) against the Homo sapiens SwissProt database (version from 23.01.2019) with the corresponding digestion settings for each measurement file. The identification result was adjusted to 5% FDR on the peptide level and 1 unique peptide per protein on a protein level. The mass spectrometry proteomics data have been deposited to the Proteomexchange Consortium via the PRIDE partner repository with the dataset identifier PxD036724 (Perez‐Riverol et al., 2019).

### 4.10 Data extraction

Filaggrin expression data was extracted from Human Protein Atlas version: 21.1 (available at http://www.proteinatlas.org; v21.1.proteinatlas.org), from the record:

https://www.proteinatlas.org/ENSG00000143631‐flg and images available at:

https://www.proteinatlas.org/ENSG00000143631‐flg/single+cell+type; https://www.proteinatlas.org/ENSG00000143631‐flg/single+cell+type/skin; https://www.proteinatlas.org/ENSG00000143631‐flg/blood+protein.

### 4.11 STRING and ubiquitination modelling

Vesicle‐related proteins list was derived from ‘Cargo recognition and sorting’ list found at Reactome database (Fabregat et al., 2017). *S. aureus*‐relevant PRRs and profilaggrin/filaggrin‐processing enzymes were identified by literature search. The compiled protein lists are included in Table S1. Proteins were subjected to the analysis with STRING database, version 11.5 (https://doi.org/10.1093/nar/gkaa1074).

Proteins of the query were subjected to the analysis with Protein‐protein interaction networks functional enrichment analysis STRING database (version 11.5) (Szklarczyk et al., 2019). Networks were generated as full STRING networks using confidence mode of display of network edges. Only text mining, experiments and databases served as sources of interactions between proteins with at least medium confidence interaction score (0.4). No expansion of the networks was applied. Generated networks were then graphically adjusted using Cytoscape software platform (version 3.9.0) (Shannon et al., 2003).

Modelling of ubiquitination was carried out based on the amino acid sequence of human profilaggrin (NP_002007.1, wild type). The sequence was examined for the presence Lys residuestab (K) as possible ubiquitination sites on individual domains and known enzyme cleavage sites.

### 4.12 Data analysis

Statistical analysis of quantitative data was carried out in GraphPad Prism v. 9.3.1 using unpaired *t*‐test, paired *t*‐test or one‐way ANOVA with Dunnett's, Šidák's or Holm‐Šidák's corrections as determined by the software based on the data characteristics and spread; *p < 0.05, **p < 0.01, ***p < 0.001, ****p < 0.0001.

## AUTHOR CONTRIBUTIONS

**Adrian Kobiela**: Data curation; Formal analysis; Investigation; Methodology; Validation; Visualization; Writing—original draft; Writing—review & editing. **Lilit Hovhannisyan**: Formal analysis; Investigation; Methodology; Validation; Visualization; Writing—review & editing. **Paulina Jurkowska**: Data curation; Investigation. Jorge **Bernardino de la Serna**: Data curation; Formal analysis; Funding acquisition; Investigation; Methodology; Software; Validation; Visualization; Writing—original draft; Writing—review & editing. **Aleksandra Bogucka**: Data curation; Formal analysis; Investigation; Methodology; Validation; Writing—review & editing. **Milena Deptuła**: Investigation; Methodology; Writing—review & editing. **Argho Aninda Paul**: Formal analysis; Visualization; Writing—review & editing. **Kinga Panek**: Formal analysis; Visualization; Writing—review & editing. **Ewa Czechowska**: Visualization; Writing—review & editing. **Michał Rychłowski**: Investigation; Writing—review & editing. **Aleksandra Królicka**: Investigation; Methodology; Resources; Writing—review & editing. **Jacek Zieliński**: Investigation; Resources; Writing—review & editing. **Susanne Gabrielsson**: Formal analysis; Methodology; Validation; Writing—review & editing. **Michał Pikuła**: Methodology; Resources; Validation; Writing—review & editing. **Magdalena Trzeciak**: Investigation; Methodology; Project administration; Resources; Writing—review & editing. **Graham S Ogg**: Conceptualization; Formal analysis; Funding acquisition; Investigation; Methodology; Project administration; Supervision; Validation; Writing—review & editing. **Danuta Gutowska‐Owsiak**: Conceptualization; Data curation; Formal analysis; Funding acquisition; Investigation; Methodology; Project administration; Supervision; Validation; Visualization; Writing—original draft; Writing—review & editing.

## CONFLICT OF INTEREST STATEMENT

The authors declare no conflict of interest in relation to this study.

## Supporting information

Supporting Information

## Data Availability

Datasets analysed in this study are available in online repositories as indicated below: https://www.ebi.ac.uk/pride/archive/, PXD036724.
