# Supplementary material for: Excess filaggrin in keratinocytes is removed by extracellular vesicles to prevent premature death and this mechanism can be hijacked by Staphylococcus aureus in a TLR2‐dependent fashion
Source: J Extracell Vesicles. 2023 Jun 20;12(6):12335. doi: 10.1002/jev2.12335 (PMC10281372; doi:10.1002/jev2.12335)
Supplement: Supplementary file 1 — Supporting Information [file JEV2-12-12335-s001.docx]

**SUPPLEMENTARY MATERIAL**


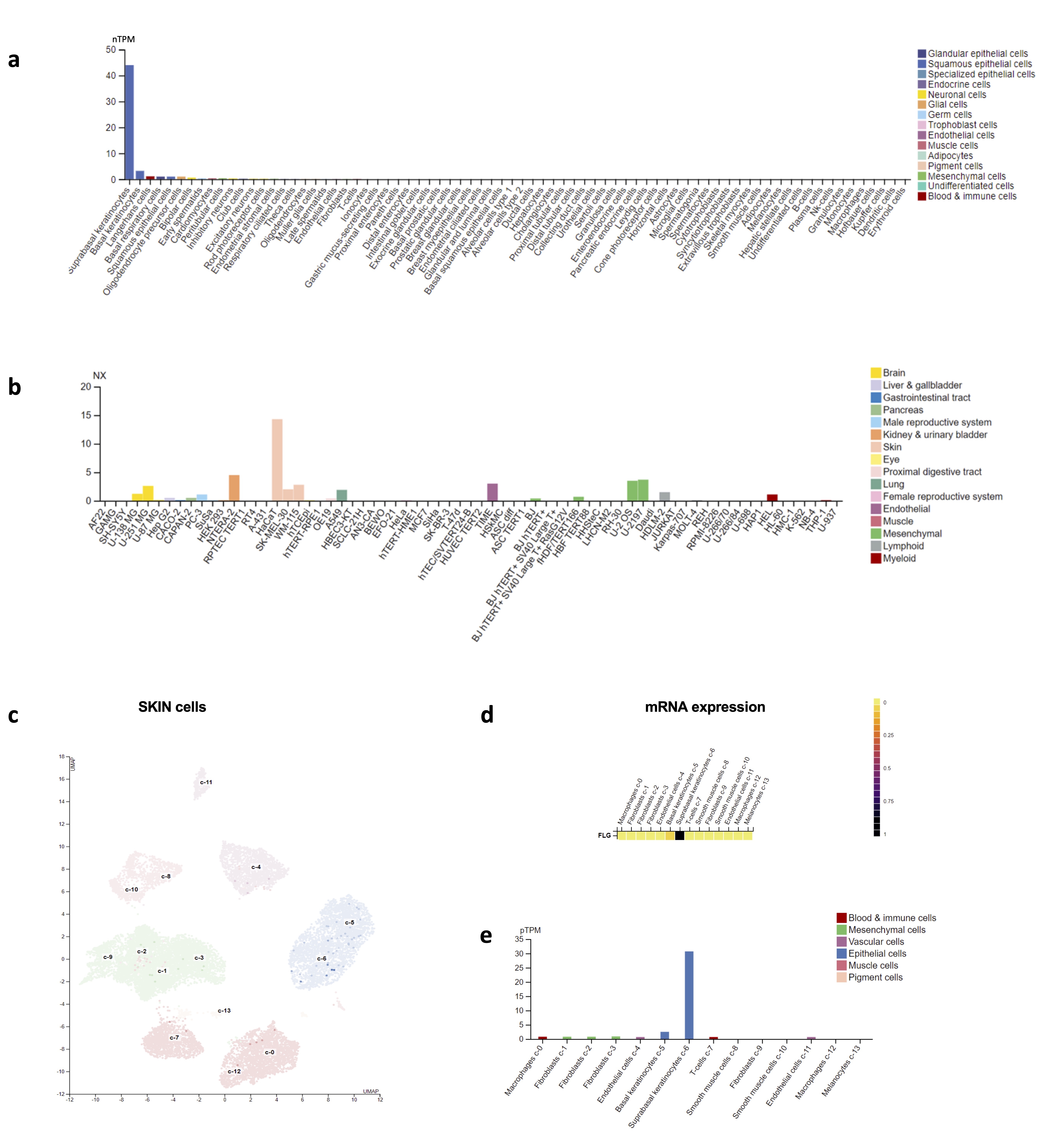


**Figure S1.**
**a)-e)** *FLG* mRNA transcript expression in **a)** primary cells, **b)** cell lines, **c)-e)** skin cells; data extracted from ProteinAtlas.


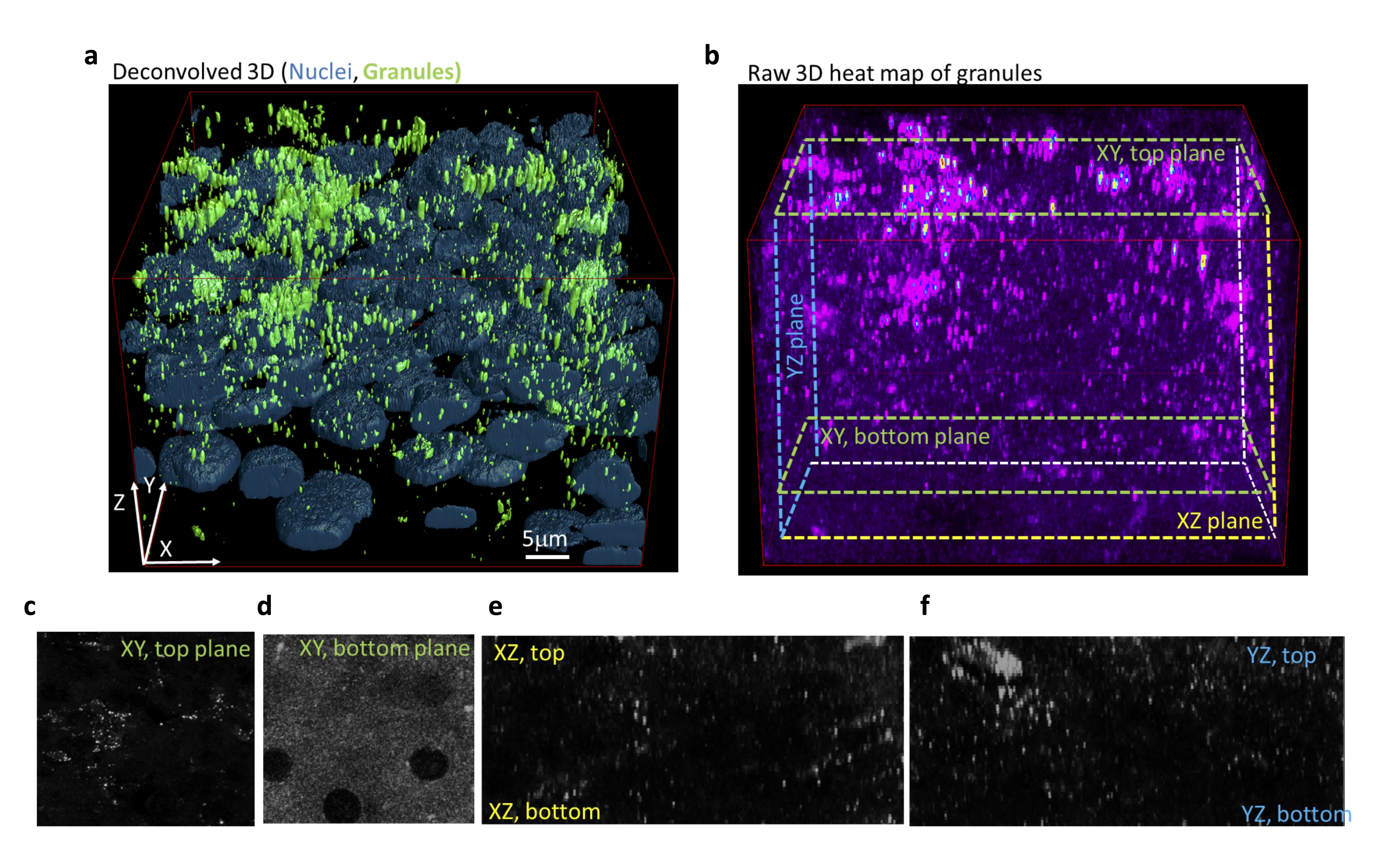


**Figure S2. Representative image of the epidermal sheet.**

**a)** deconvolved image, indicating KHG accumulation; **b)** raw 3D heat map visualizing KHGs representative cross-section of epidermal sheet with indication of the planes; **c)** XY top plane (predominantly granular staining); **d)** XY bottom plane (dispersed/filamentous staining); **e)** XZ cross-section; **f)** YZ cross-section; n=6; scale bar 5 µm.


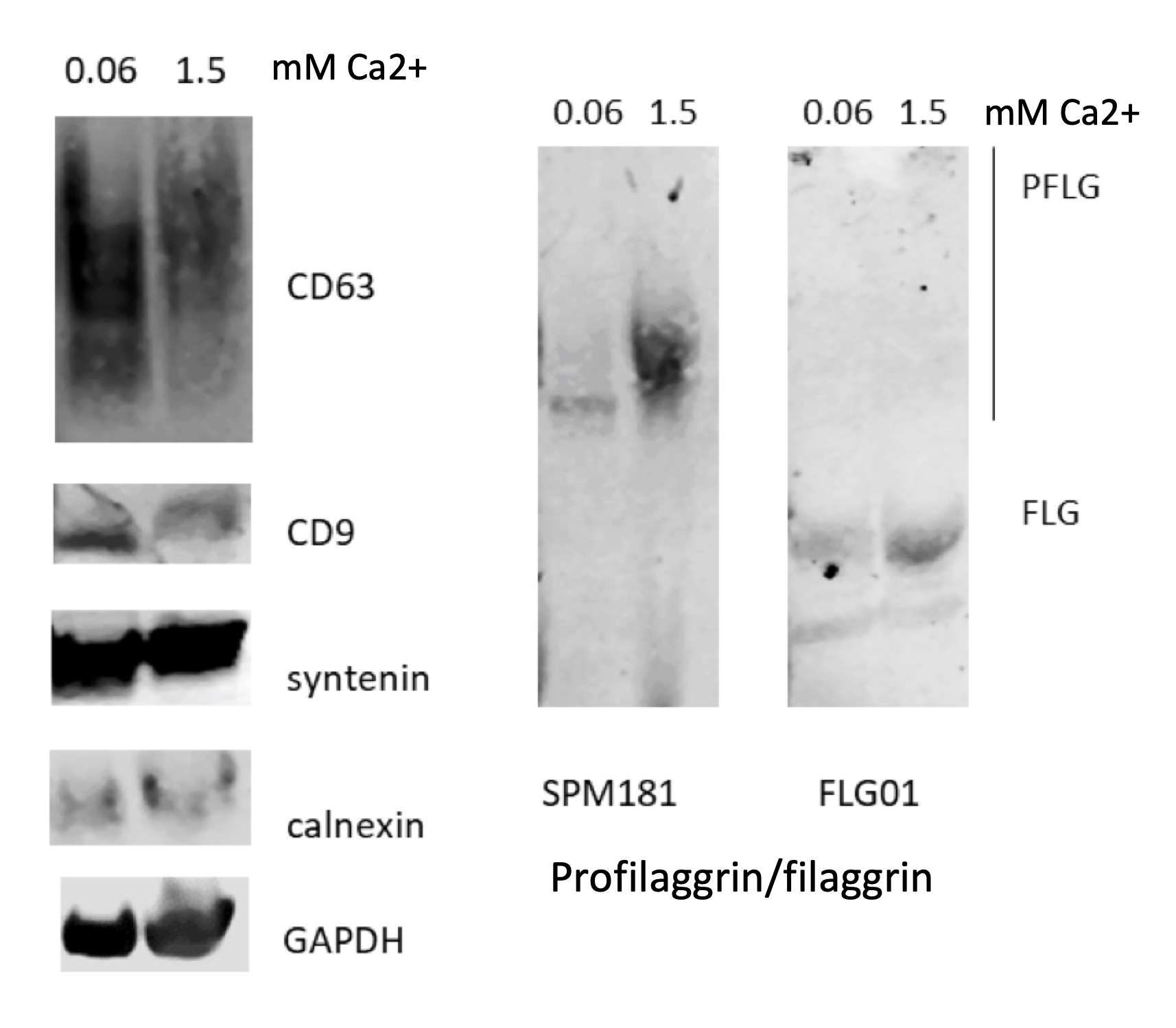


**Figure S3.**

Identification of exosomal markers and profilaggrin/filaggrin products in lysates of primary keratinocytes (NHEK) cultured in the presence of 0.06 mM Ca^2+^ and 1.5 mM Ca^2+^ by western blot.


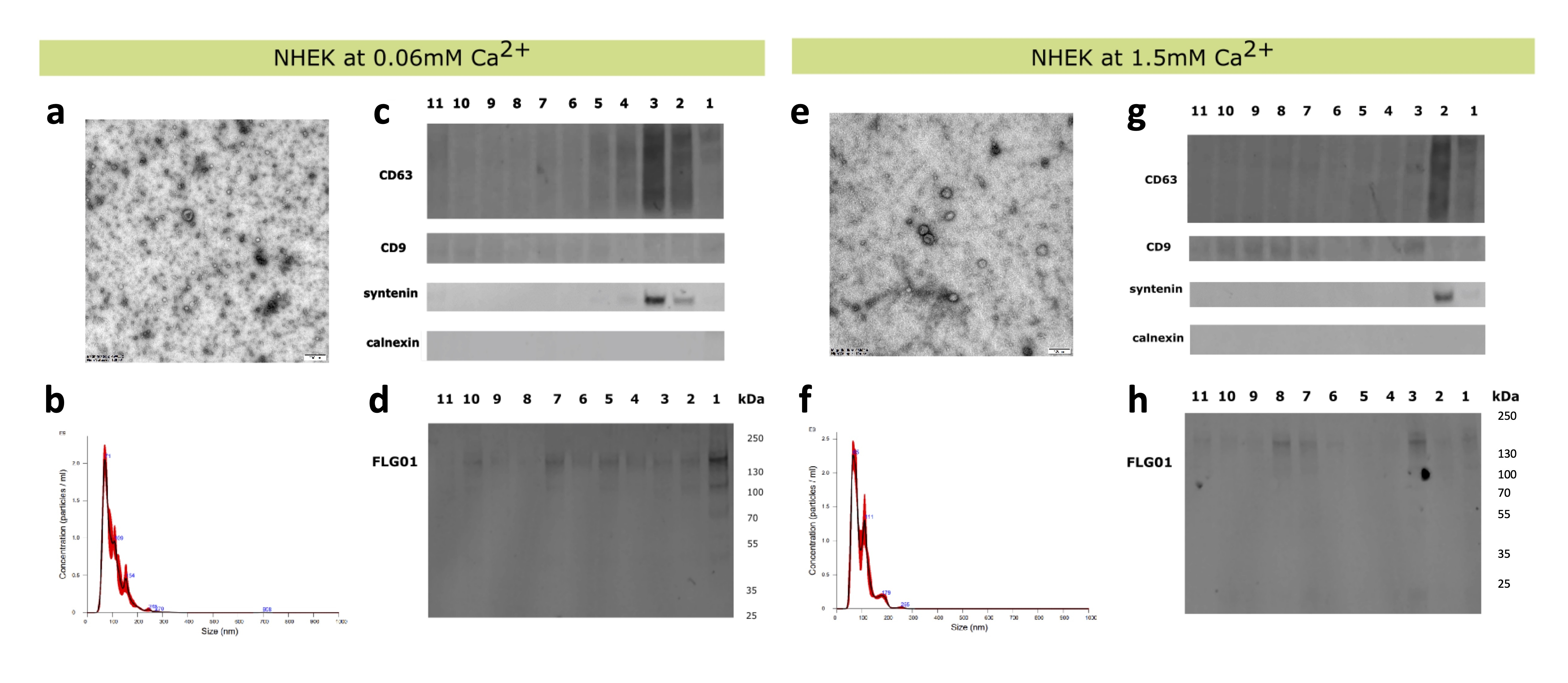


**Figure S4.**

**a)** confirmation of the typical sEV morphology by electron microscopy and **b)** sEV size distribution by Nanoparticle Tracking analysis (NTA) of sEVs (100K pellet) secreted by primary keratinocytes cultured in the presence of 0.06 mM Ca^2+^ (NHEK_0.06_); **c)-d)** detection of **c)** exosomal markers and **d)** profilaggrin/filaggrin products in fractions collected following the iodixanol/sucrose gradient purification of NHEK_0.06_-produced sEV by western blot; **e)** confirmation of the typical sEV morphology by electron microscopy and **f)** sEV size distribution by Nanoparticle Tracking analysis (NTA) of sEVs (100K pellet) secreted by primary keratinocytes cultured in the presence of 1.5 mM Ca^2+^ (NHEK_1.5_);
**g)-h)** detection of **g)** exosomal markers and **h)** profilaggrin/filaggrin products in fractions collected following the iodixanol/sucrose gradient purification of NHEK_1.5_-produced sEV by western blot, example donor with detectable signal in sEVs, n=3 donors.


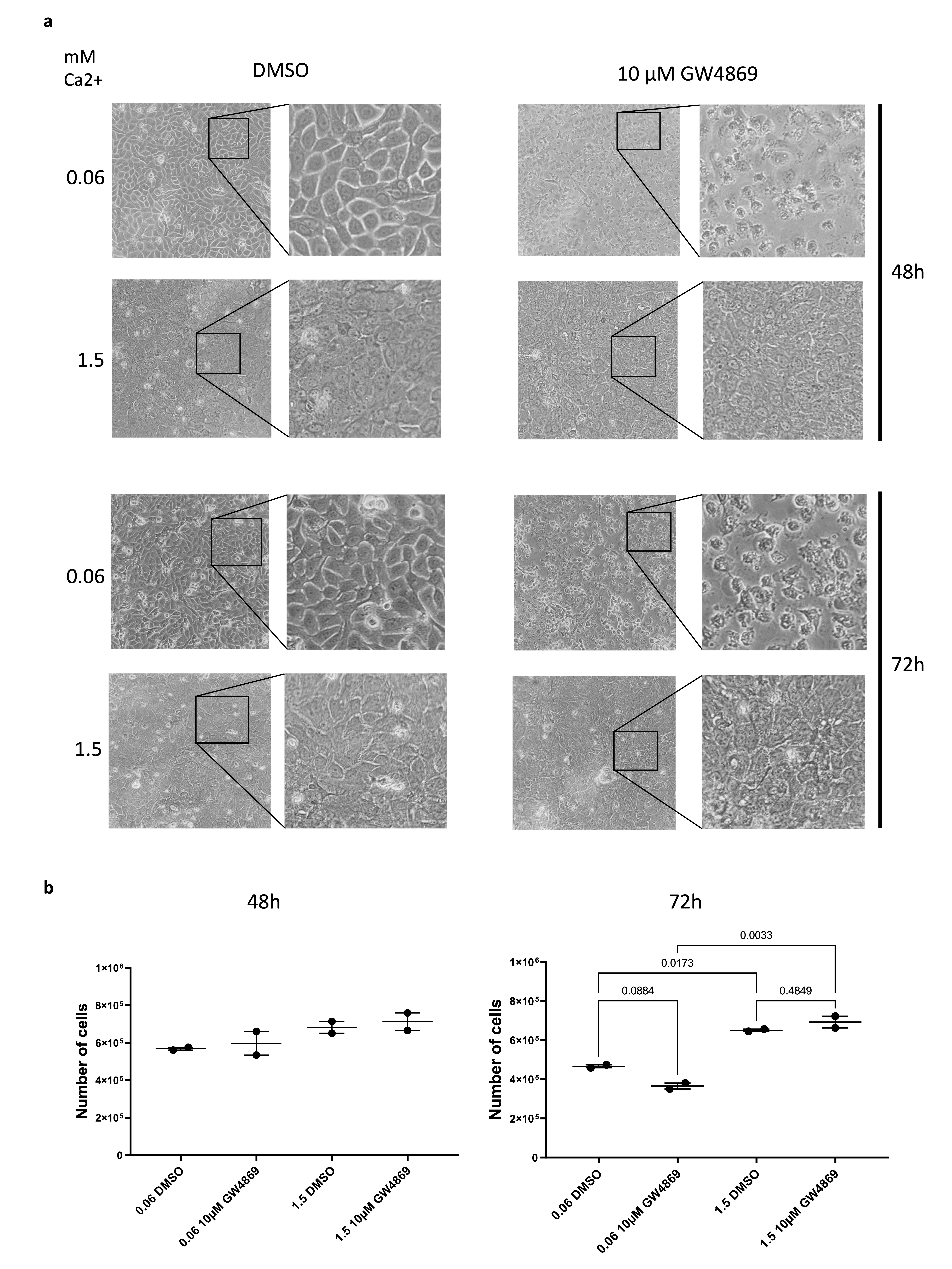


**Figure S5.**

N/TERT-1 cells were cultured in the presence of 0.06 and 1.5 mM Ca^2+^ and treated with 10 µM of GW4869, an inhibitor of exosome biogenesis/release for 48 or 72 h; **a)** example bright field microscopy images following incubation; **b)** number of live cells harvested after treatment; means +/- SEM from n=2 biological replicates are shown; one-way ANOVA with Tukey’s multiple comparisons test.


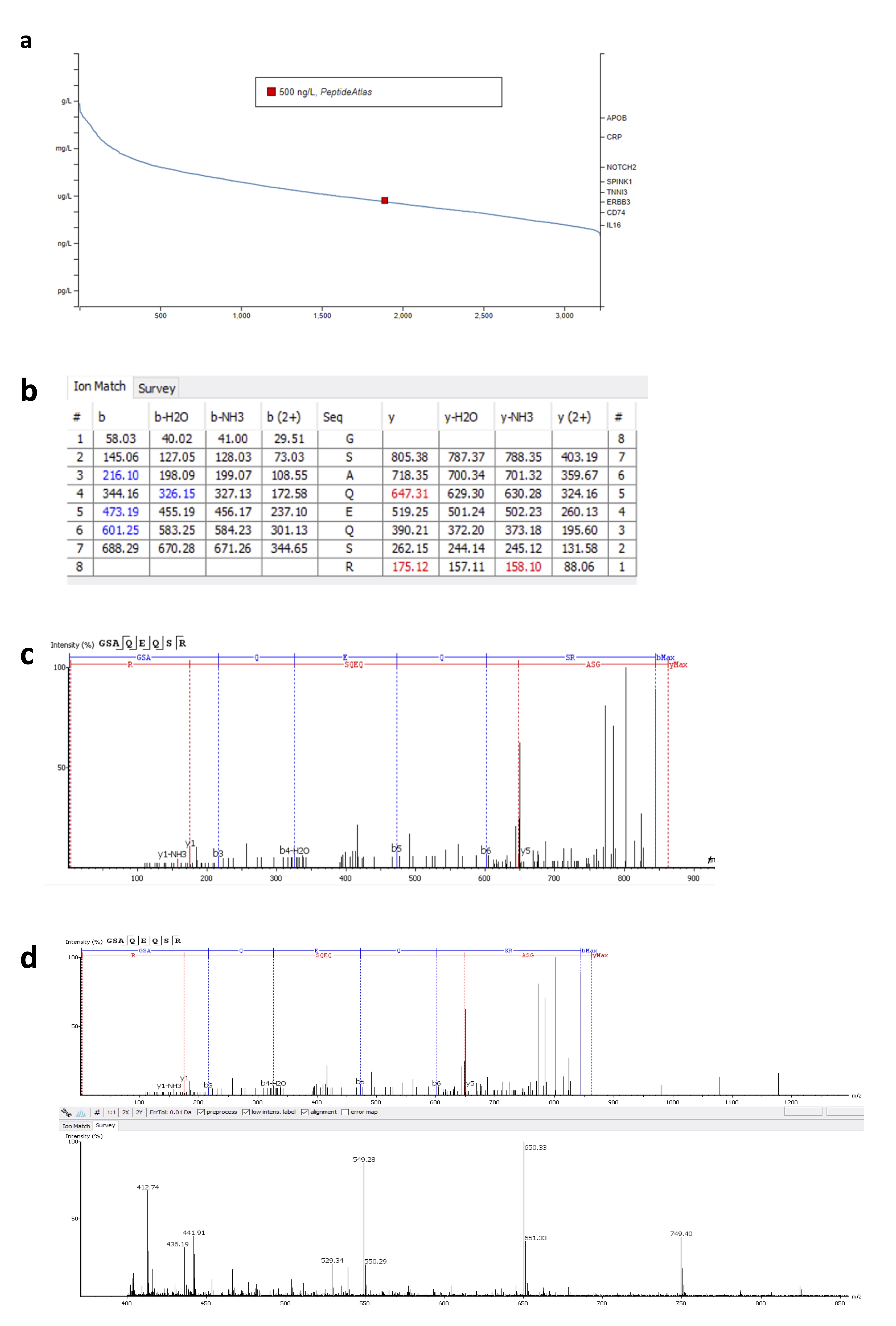


**Figure S6.**

**a)** detection of the filaggrin protein in human blood plasma; data from the Protein Atlas; **b)** intensities and **c)-d)** MS/MS spectra of filaggrin-relevant peptides detected in human blood plasma-derived sEVs (100k pellet); pooled samples from n=3 donors.


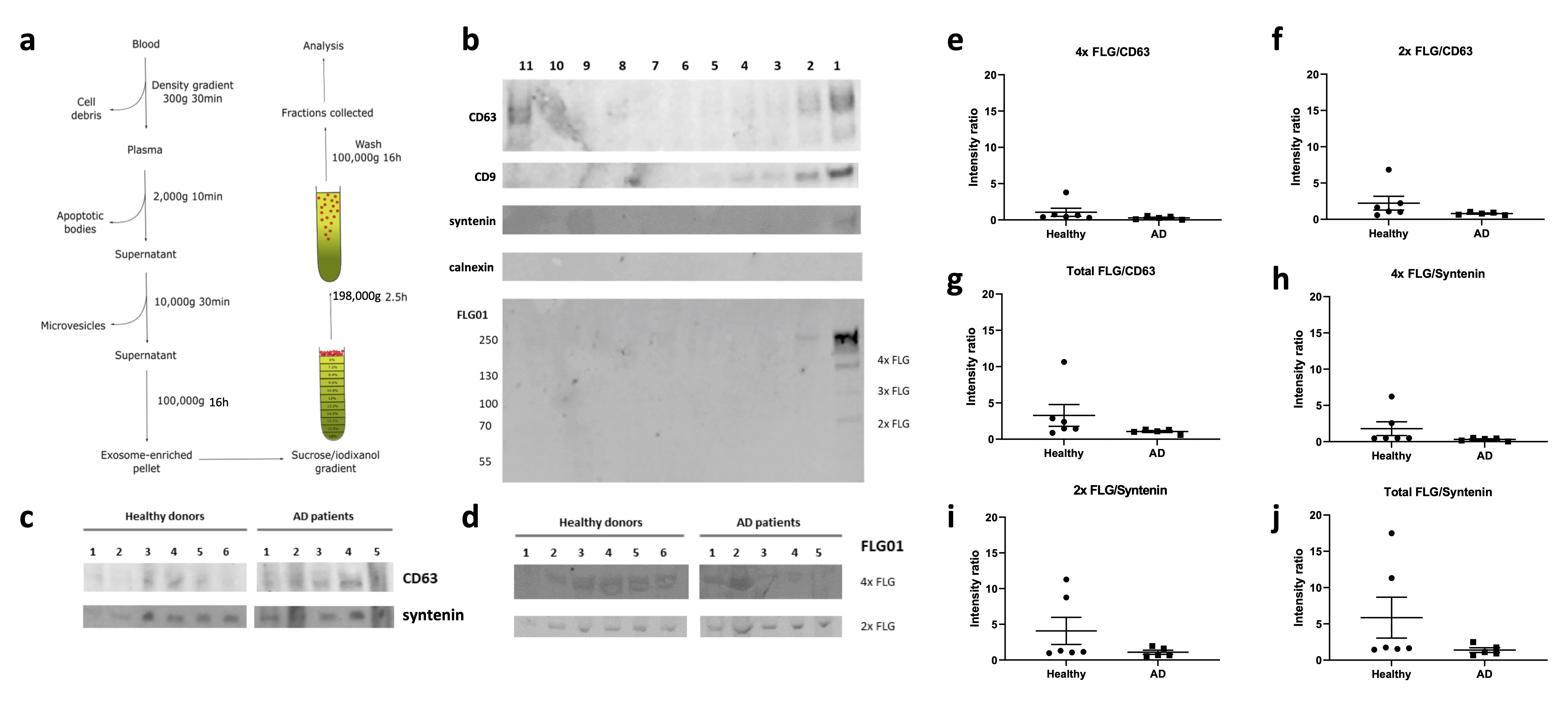


**Figure S7.**

**a)** protocol for isolation and purification of human blood plasma-derived sEVs; **b)** identification of exosomal markers and profilaggrin/filaggrin products in fractions collected after the iodixanol/sucrose gradient purification of human blood plasma-derived sEV/exosomes; **c)-d)** detection of **c)** exosomal markers and **d)** profilaggrin/filaggrin products in sEVs (100K pellet; sucrose/iodixanol-purified) from blood plasma of healthy individuals and AD patients; **e)-j)** analysis of the filaggrin-relevant signal in relation to exosome markers **e)-g)** CD63 and **h-j)** syntenin in sEVs (100K pellet) isolated from blood plasma of healthy individuals and AD patients detected by western blot; combined data from n=6 healthy donors and n=5 AD patients, means +/- SEM are shown, unpaired t-test, data from one of the assessed AD patient were excluded from the analysis due to high background signal.


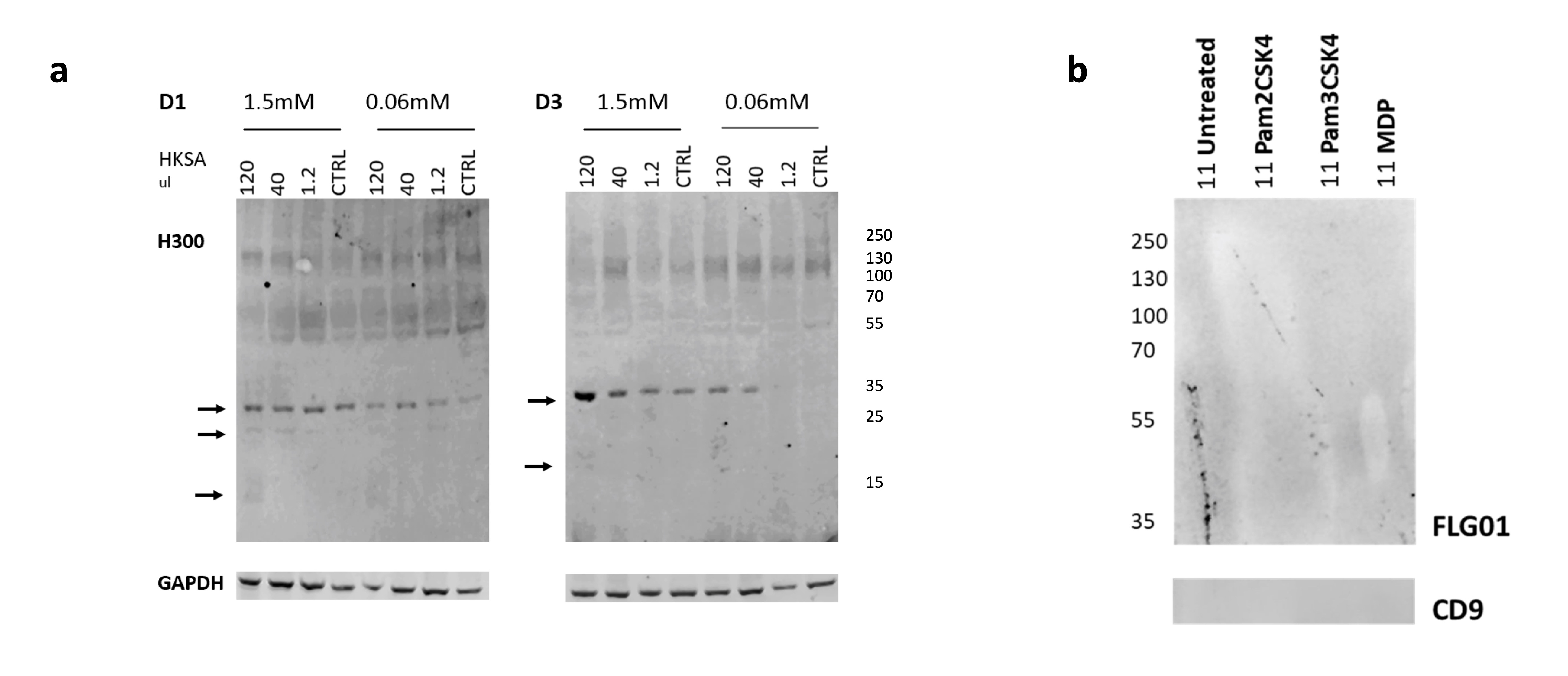


**Figure S8.**

**a)** detection of profilaggrin/filaggrin signal in lysates of heat-killed *S. aureus* (HKSA)-treated primary keratinocytes on day 1 and day 3 post-treatment, GAPDH was used as a loading control; n=2; **b)** detection of profilaggrin/filaggrin and CD9 signal in fractions ‘11’ collected after iodixanol/sucrose gradient purification of sEV/exosomes produced by N/TERT-1 cells stimulated by TLR2 and NOD2 agonists by western blot, example data from n=3 biological replicates shown; Pam2CSK4, TLR2/6 agonist; Pam3CSK4, TLR2/1 agonist; MDP, NOD2 agonist.


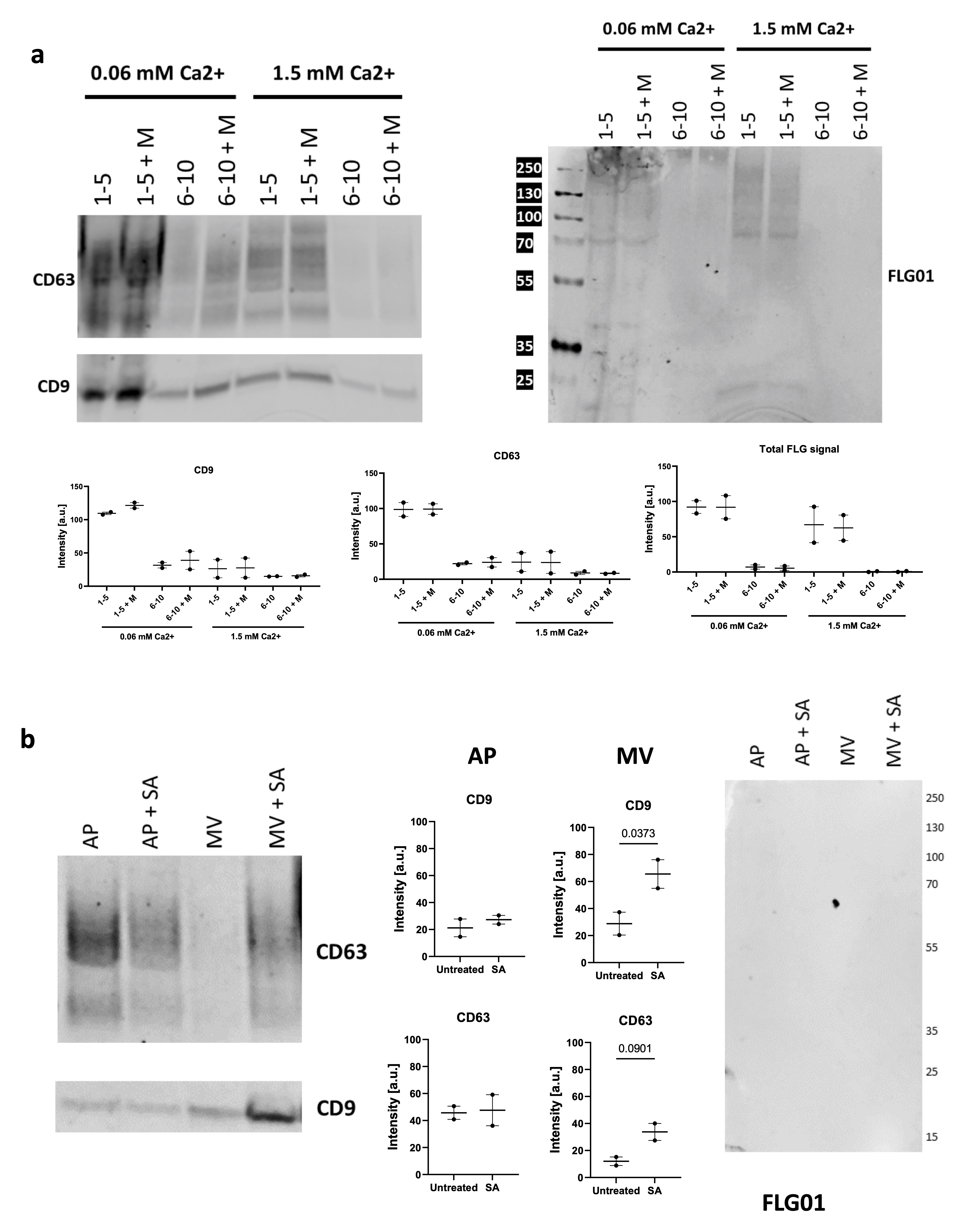


**Figure S9.**

**a)** detection of profilaggrin/filaggrin and exosomal marker signal in in iodixanol/sucrose gradient fractions following purification of sEV/exosomes produced by N/TERT-1 cells treated with uninoculated *S. aureus* growth medium by western blot; example images of membranes from n=2 biological replicates shown; quantified data are means +/- SEM; one-way ANOVA with Šídák's multiple comparisons test; **b)** detection of profilaggrin/filaggrin and marker signal in apoptotic bodies and microvesicles produced by N/TERT-1 cells grown in the presence of 1.5 mM Ca^2+^ and treated with *S. aureus* growth medium; example images of membranes from n=2 biological replicates shown; quantified data are means +/- SEM; paired t-test. M, treatment with uninoculated *S. aureus* growth medium; AP, apoptotic bodies; MV, microvesicles; SA, treatment with *S. aureus* growth medium.


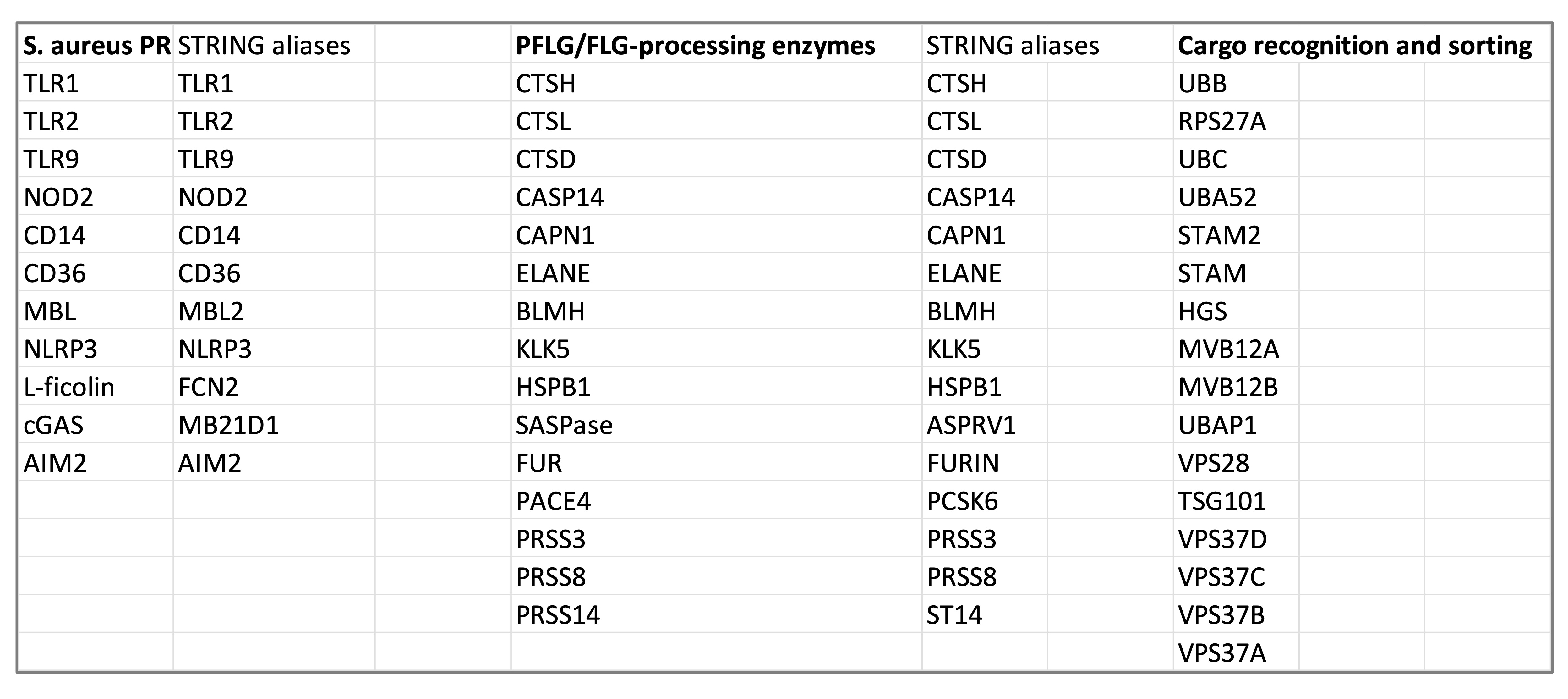


**Table S1.** A list of proteins identified by Reactome as those related to “cargo recognition and sorting” and used for STRING analysis.


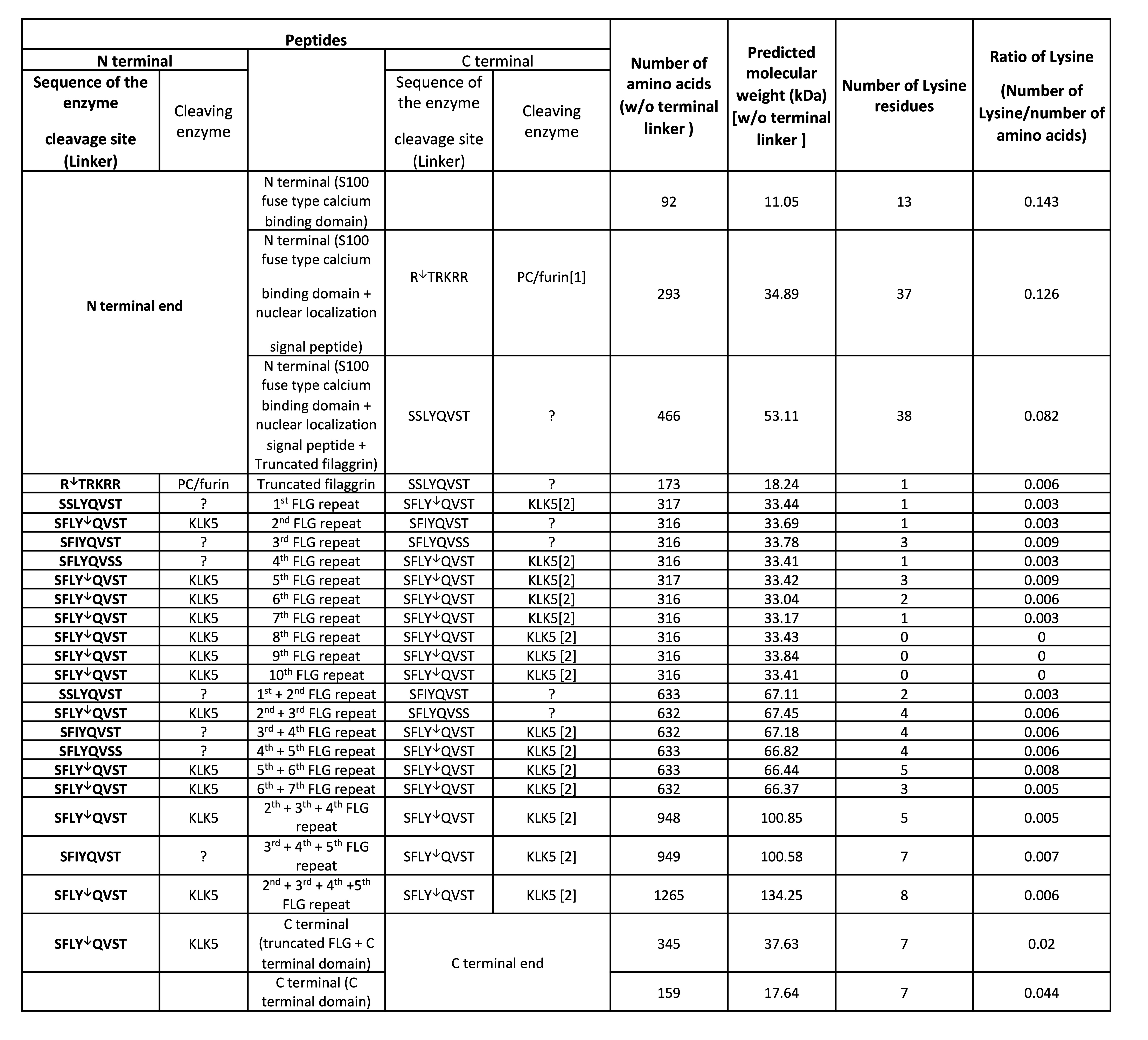


**Table S2.** Products of enzymatic cleavage of profilaggrin and their relative Lys content.


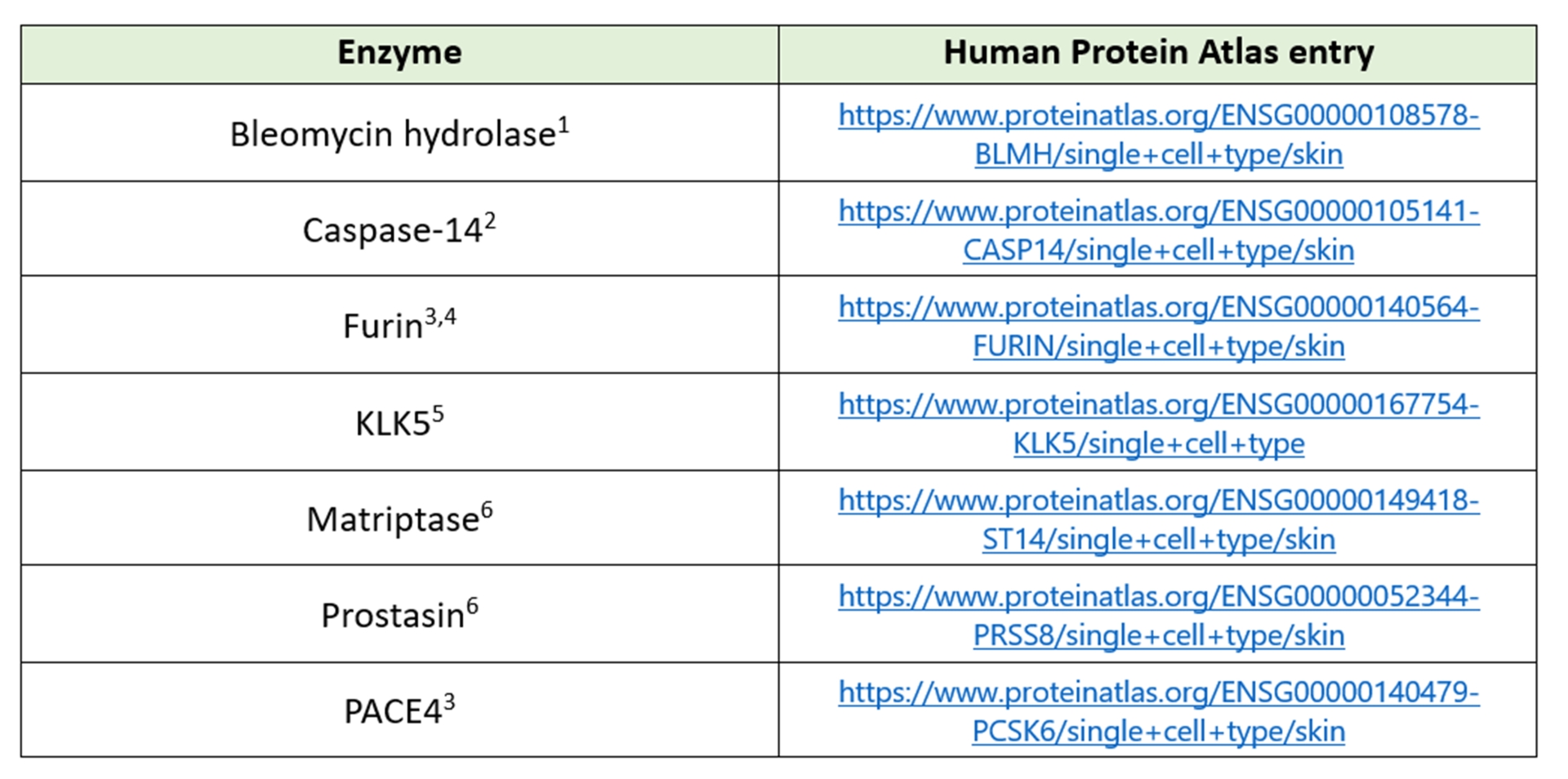


**Table S3.** Profilaggrin/filaggrin processing enzymes expressed in 2D keratinocyte cultures and basal keratinocytes. KLK5, kallikrein 5; PACE4, paired basic amino acid cleaving enzyme 4.

**Supplementary references**

1. Gutowska-Owsiak, D. et al. Histamine exerts multiple effects on expression of genes associated with epidermal barrier function. *J. Investig. Allergol. Clin. Immunol.* **24,** 231-239 (2014).
2. Kuechle, M. K., Predd, H. M., Fleckman, P., Dale, B. A. & Presland, R. B. Caspase-14, a keratinocyte specific caspase: mRNA splice variants and expression pattern in embryonic and adult mouse. *Cell Death Differ. 2001* **8**, 868–870 (2001).
3. Pearton, D. J., Nirunsuksiri, W., Rehemtulla, A., Lewis, S. P., Presland, R. B., Dale, B. A. Proprotein convertase expression and localization in epidermis: evidence for multiple roles and substrates. *Exp. Dermatol.* **10**, 193–203 (2001).
4. Spencer, J. D., Gibbons, N. C. J., Böhm, M. & Schallreuter, K. U. The Ca2+-binding capacity of epidermal furin is disrupted by H2O2-mediated oxidation in vitiligo. *Endocrinology* **149**, 1638–1645 (2008).
5. Williams, M. R., Nakatsuji, T., Sanford, J. A., Vrbanac, A. F. & Gallo, R. L. Staphylococcus aureus Induces increased serine protease activity in keratinocytes. *J. Invest. Dermatol.* **137,** 377–384 (2017).
6. Alef, T. et al. Ichthyosis, follicular atrophoderma, and hypotrichosis caused by mutations in ST14 is associated with impaired profilaggrin processing. *J. Invest. Dermatol.* **129**, 862–869 (2009).
